# Intrinsic units: identifying a system’s causal grain

**DOI:** 10.1093/nc/niag013

**Published:** 2026-04-15

**Authors:** William Marshall, Graham Findlay, Larissa Albantakis, Giulio Tononi

**Affiliations:** Department of Mathematics and Statistics, Brock University, 1812 Sir Isaac Brock Way, St. Catharines, Ontario, L2S 3A1, Canada; Department of Psychiatry, University of Wisconsin-Madison, 6001 Research Park Blvd, Madison, WI, 53719, United States; Department of Psychiatry, University of Wisconsin-Madison, 6001 Research Park Blvd, Madison, WI, 53719, United States; Department of Psychiatry, University of Wisconsin-Madison, 6001 Research Park Blvd, Madison, WI, 53719, United States

**Keywords:** theories and models, philosophy, consciousness, computational modeling, integrated information, causal emergence

## Abstract

Integrated information theory (IIT) aims to account for the quality and quantity of consciousness in physical terms. According to IIT, a substrate of consciousness must be a system of units (e.g. synapses, neurons, minicolumns, etc.) that is a maximum of intrinsic, specific, unitary cause-effect power, quantified by integrated information ($\varphi _{s}$). The grain of each unit must be the one—from micro (finer) to macro (coarser)—that maximizes the system’s integrated information. Here we provide a framework for computing the integrated information of systems whose constituents include macro units, and in doing so provide the means to identify a system’s *intrinsic units*—those that constitute the system from its intrinsic perspective, and directly account for its experience. First, we formalize what it means for these units, as part of a substrate of consciousness, to satisfy IIT’s postulates of physical existence. Next, we extend the mathematical framework of IIT 4.0 to assess cause-effect power across grains. Then, using simple, simulated systems, we show that the integrated information of systems containing macro units can be higher than that of corresponding systems of micro units. Three examples highlight specific kinds of macro units, and how each kind can increase cause-effect power. The implications of the framework are discussed in the broader context of IIT, including how it provides a foundation for tests and inferences about consciousness.

## Introduction

One goal of the scientific study of consciousness is to ascertain its neural substrate. Much attention has been given to the question of which regions of the brain support consciousness (Boly *et al.* 2017; Odegaard *et al.* 2017). No less important, but less often considered, is the question of the units constituting the substrate of consciousness and their “grain.” Are the units individual neurons, synapses, groups of neurons, or the smallest units that we can possibly manipulate and observe? Is a unit’s state over a hundred milliseconds, or one millisecond, or one second what matters for consciousness? These issues are not only empirical but also call for a theoretical understanding of why certain brain regions qualify as a substrate of consciousness, while others do not, and why the grain of each unit within a substrate is what it is.

Integrated information theory (IIT) aims to account for consciousness—its quality and quantity—by starting from phenomenology and identifying its essential properties—the *axioms* of phenomenal existence—that are true of every conceivable experience: *existence*, *intrinsicality*, *information*, *integration*, *exclusion*, and *composition* (Albantakis *et al.* 2023b).

The axioms of phenomenal existence are formulated as corresponding physical properties, called *postulates*, that must be satisfied by the substrate of consciousness. Physical existence is defined operationally in terms of cause-effect power, and the postulates therefore require that a substrate of consciousness have cause-effect power (existence) upon itself (intrinsicality), in a way that is specific (information), unitary (integration), definite (exclusion), and structured (composition). In principle, by evaluating whether and in what way a candidate substrate satisfies all of the postulates, one can evaluate whether and in what way it is conscious, with no additional ingredients.

IIT provides a precise mathematical definition of intrinsic, specific, unitary, definite, and structured cause-effect power, so that it is possible (in principle) to assess whether any given physical system satisfies the postulates. The *integrated information* ($\varphi _{s}$) of a system quantifies the extent to which a set of units in a state possesses intrinsic, specific, unitary cause-effect power. According to IIT, a substrate of consciousness, called a *complex*, is then a set of units in a state with greater $\varphi _{s}$ than all overlapping systems, including those at different grains (Albantakis *et al.* 2023b). Because a complex is a maximum of integrated information, it is always definite—there is always a sufficient reason (maximization of $\varphi _{s}$) why it has precisely the borders and constituents that it does, and why its units have their particular grains.

The IIT framework provides both the means to identify substrates of consciousness—by finding maxima of integrated information—and to account for “what it is like” to be those substrates—by *unfolding* their cause-effect structures. Unfolding, though a crucial aspect of IIT, plays no role in assessing the cause-effect power of macro systems. For this reason, we will not discuss unfolding further, and refer interested readers to (Haun and Tononi, 2019; Marshall *et al.* 2023; Albantakis *et al.* 2023b; Comolatti and Hoel, 2025).

Initial work towards determining the grain of a substrate’s units introduced the notion of *micro units*—the finest units that can be observed and manipulated—and *macro units*—coarser units derived from sets of micro units. This work demonstrated that the cause-effect power of a system, as measured by either effective information (Tononi and Sporns, 2003) or integrated information (Hoel *et al.* 2013

2016; Marshall *et al.* 2018), could peak when constituted of macro units. Subsequent work has further explored how and why such “causal emergence” is possible, across a wide variety of fields and measures (Comolatti *et al.* 2025).

IIT’s framework has been refined over time (Tononi, 2004; Balduzzi and Tononi, 2008; Oizumi *et al.* 2014; Albantakis *et al.* 2023b), and includes several recent developments (Haun and Tononi, 2019; Barbosa *et al.* 2021; Marshall *et al.* 2023) (for applications outside of consciousness science, see (Albantakis *et al.* 2014; Marshall *et al.* 2017; Albantakis *et al.* 2019)). The current framework—IIT 4.0—aims to provide a complete, self-consistent formulation of the postulates in mathematical terms, guided by clearly articulated ontological and methodological principles (Albantakis *et al.* 2023b).

The goal of the current work is to provide a means of identifying the grain of a system’s units, and for computing the integrated information of systems whose constituents include macro units, in a way that is consistent with IIT’s postulates and principles. To this end, we introduce the notion of a substrate’s *intrinsic units*—those that account for its experience, whatever their grain may be—and formalize what it means for these units, as part of a substrate of consciousness, to satisfy IIT’s postulates of physical existence.

In the Theory section, we briefly review IIT 4.0’s mathematical framework for measuring the integrated information of systems of micro units, and then extend this framework to systems containing macro units. We show how $\varphi _{s}$ can be used to assess whether a single candidate unit satisfies IIT’s postulates, just as $\varphi _{s}$ can be used to assess whether a candidate substrate satisfies IIT’s postulates, with a crucial distinction: whereas a candidate substrate’s $\varphi _{s}$ must exceed that of any overlapping system (i.e. any system constructed from a subset, superset, or paraset of the candidate substrate’s units), a candidate unit’s $\varphi _{s}$ need only exceed that of its subsets. This distinction reflects the key difference that, for a candidate system, the postulates are requirements for consciousness, whereas for a candidate unit, they are simply a logical prerequisite for being a part of a complex. In the Examples section, the updated framework is applied to simple systems, demonstrating how and why macro-grain systems can have higher $\varphi _{s}$ than their corresponding micro-grain systems, and when one should expect to find systems with intrinsic units of different grains in practice. Finally, in the Discussion section, we provide a brief discussion of the importance of this framework for future work.

## Theory

In this section, we first highlight features of IIT’s mathematical framework that are necessary for its extension to macro grains. Next, we introduce a definition for macro units constituted of micro units and extend the formalization of IIT’s postulates to systems containing macro units by introducing the notion of intrinsic units. Finally, we extend the mathematical framework so it can be used to measure the cause-effect power of systems containing macro units.

### Cause-effect power at the micro grain

According to IIT, something can be said to exist physically if it can “take and make a difference.” (i.e. bear a cause and produce an effect). Operationally, it must be possible to manipulate the system’s units (change their state) and observe the result.

The starting point of IIT’s mathematical framework is a discrete-valued stochastic model for a physical universe $U = \{U_{1}, \ldots , U_{n}\}$ of $n$ interacting units with state space $\Omega _{U} = \{0, 1\}^{n}$. We use lowercase to indicate the state of a set of units, e.g. $u, u^{\prime} \in \Omega _{U}$. Occasionally, we will use lowercase letters to indicate a set of units in a state, for example, $s \subseteq u$, which should be interpreted as $\{S = s\}$ being a subset of $\{U = u\}$. While this usage is a slight abuse of notation, it greatly reduces notational burden. Because the physical existence of $U$ is formulated operationally as cause-effect power, $U$ is defined by its potential interactions, assessed in terms of conditional probabilities. We denote the complete *transition probability matrix* (TPM) of a universe $U$ over a system update $u \rightarrow u^{\prime}$ as:


(1) \begin{align*}& \mathcal{T}_{U} \equiv p(u^{\prime} \mid u) \qquad u^{\prime}, u \in \Omega_{U}.\end{align*}

The TPM provides a complete description of $U$ at the micro grain—the finest grain at which manipulation and observation is possible, with nothing omitted from the causal model—which means that we can determine the conditional probabilities in (1) for every system state, with $p(u^{\prime} \mid u) = p(u^{\prime} \mid \operatorname{do}(u))$, where the “do-operator” $\operatorname{do}({u})$ indicates that $u$ is imposed by intervention (Pearl, 2000; Ay and Polani, 2008; Janzing *et al.* 2013; Albantakis *et al.* 2019). This implies that $U$ corresponds to a complete causal network (Albantakis *et al.* 2019).

According to IIT, $\mathcal{T}_{U}$ does not merely *describe* the physical universe, but rather *is* the physical universe, because there is no need to posit primitive categorical properties like mass, charge, or spin that would “underlie” or come prior to $\mathcal{T}_{U}$—there is just cause-effect power. What a micro physical system is, is just its TPM, and vice versa (Albantakis *et al.* 2023b; Chis-Ciure 2024a).

Because $U$ is assumed to be a complete causal network, it will not exhibit “instantaneous causation.” More formally, the individual random variables $U_{i} \in U$, conditional on the preceding state of $U$, are independent from each other:


(2) \begin{align*}& p(u^{\prime} \mid u) = \prod_{i=1}^{n} p(u^{\prime}_{i} \mid u), \qquad u, u^{\prime} \in \Omega_{U}.\end{align*}

For an extension of IIT to quantum systems, where model completeness may not imply conditional independence between units, see (Albantakis *et al.* 2023a).

*Micro units* are the finest units that can be observed and manipulated, satisfying the minimal requirements for cause-effect power (i.e. physical existence) (Albantakis *et al.* 2023b). Accordingly, micro units must have exactly two states—“this way” and “not this way.” One state is simply the complement of the other, whichever state is picked, with no further qualification. Having more than two states would imply an internal mechanism distinguishing among “this way,” “that way,” and “the other way,” contradicting the claim that these are the finest units.

For any *candidate substrate* (also called a *candidate system*) $S \subseteq U$ in a state $s \subseteq u$, the IIT 4.0 framework defines its *system integrated information*  $\varphi _{s}(s)$ (Marshall *et al.* 2023; Albantakis *et al.* 2023b). Based on the postulates of intrinsicality, information, and integration, $\varphi _{s}(s)$ quantifies how the system specifies a *cause-effect state* as a whole, above and beyond how it specifies the same cause-effect state as independent parts (Albantakis *et al.* 2023b). Per the *principle of minimal existence*, which states that “nothing exists more than the least it exists” (e.g. “a chain is only as strong as its weakest link”), the comparison between the whole and its parts is performed by partitioning the system and evaluating the impact of the *minimum partition*—the partition over which the system is least irreducible (Albantakis *et al.* 2023b). The system integrated information, $\varphi _{s}$, is defined as the *intrinsic information* of the whole (Barbosa *et al.* 2020

2021), relative to the parts specified by its minimum partition. We do not present the full definition or algorithm for obtaining $\varphi _{s}$ here—only the parts that are relevant for extending the framework to macro units.

For any candidate system, $\varphi _{s}(s)$ is defined based on two system-specific transition probability matrices, $\mathcal{T}_{c}$ and $\mathcal{T}_{e}$ (for describing causes and effects respectively). The system TPMs are computed by *causally marginalizing* the units $W = U \setminus S$ conditional on the current state $u$, as described below. $W$ in state $w$ are referred to as the system’s *background units* or *background conditions*, and they may partly enable its cause-effect power (Albantakis *et al.* 2023b). $\mathcal{T}_{c}$ and $\mathcal{T}_{e}$ therefore capture intrinsic cause-effect power of the system within the context of a set of background conditions.

To causally marginalize background units, we evaluate the likelihood of each possible background state $w$, by computing the conditional distribution $q(w \mid u)$ for the state of the background units conditional on the current state of $U$. For evaluating effects of the current state, the relevant state of the background is its current state, which is fully determined by the current state of the universe:


(3) \begin{align*}& q_{e}(w \mid u) = \begin{cases} 1 & \text{ if} \ w = u \setminus s \\ 0 & \text{ otherwise} \end{cases}, \qquad w \in \Omega_{W}, ~ u \in \Omega_{U}.\end{align*}

For evaluating causes of the current state, the relevant state of the background is its past state. The current state (of the universe) is used to compute the probability distribution over possible past states of the background units, which is not necessarily uniform or deterministic. This distribution is computed using Bayes’ rule, assuming a uniform marginal distribution of the previous state:


(4) \begin{align*} q_{c}(w \mid u) &= \sum_{\bar{s}} p(\bar{s}, w \mid u)\qquad\qquad\qquad\ \ \ \ \end{align*}

(5) \begin{align*} &= \sum_{\bar{s}} \frac{p(u \mid \bar{s}, w)p(w, \bar{s})}{\sum_{\bar{u}}p(u \mid \bar{u})p(\bar{u})} \end{align*}

(6) \begin{align*} &\qquad\qquad\qquad\qquad\, = \frac{\sum_{\bar{s}} p(u \mid \bar{s}, w)}{\sum_{\bar{u}}p(u \mid \bar{u})}, \qquad w \in \Omega_{W}, ~ u \in \Omega_{U}. \end{align*}

The corresponding TPMs ($\mathcal{T}_{c}$ or $\mathcal{T}_{e}$) are a weighted (by $q_{c/e}(w \mid u)$) average of transition probabilities over possible states of background units. Note that transition probabilities are computed one unit at a time, and then a product is used to compute probabilities for the whole system. This removes correlations among the units introduced by a weighted average of background conditions, and restores the conditional independence property. For the cause TPM:


(7) \begin{align*}& \mathcal{T}_{c} \equiv p_{c}(s^{\prime} \mid s) = \prod_{i = 1}^{|S|} \sum_{\bar{w}} p(s^{\prime}_{i} \mid s, \bar{w})q_{c}(\bar{w} \mid u) \qquad s^{\prime}, s \in \Omega_{S}, ~ u \in \Omega_{U}.\end{align*}

For the effect TPM, the form of $q_{e}(w \!\mid \!u)$ leads to a simplified expression:


(8) \begin{align*} \mathcal{T}_{e} \equiv p_{e}(s^{\prime} \mid s) = \prod_{i = 1}^{|S|} \sum_{\bar{w}} p(s^{\prime}_{i} \mid s, \bar{w})q_{e}(\bar{w} \mid u) = \ & p(s^{\prime}\mid s, u \setminus s) \nonumber \\[-11pt] & s^{\prime}, s \in \Omega_{S}, ~u \in \Omega_{U}.\end{align*}

From $\mathcal{T}_{c}$ and $\mathcal{T}_{e}$, one can compute $\varphi _{s}(s)$ as outlined in (Albantakis *et al.* 2023b).

According to the exclusion postulate, a substrate of consciousness must be definite: there must be a reason why it consists of these units and not others. The reason is provided by the *principle of maximal existence*, which states that among competing existents, the one that actually exists is the one that exists the most. Furthermore, if maximal existence is the sufficient reason for a complex being supported by a given set of units, it is also the sufficient reason for not being supported by subsets, supersets, or parasets of that set. This implies that complexes cannot overlap, in line with the notion that a micro unit’s cause-effect power should not be counted multiple times (Tononi forthcoming).

Since existence as *one* entity is quantified by integrated information $\varphi _{s}$, complexes can be identified as maxima of $\varphi _{s}$ (Albantakis *et al.* 2023b). That is, $s$ is a complex if:


(9) \begin{align*}& s \cap s^{\prime} \neq \varnothing \Rightarrow \varphi_{s}(s)> \varphi_{s}(s^{\prime}) \qquad \forall s^{\prime} \neq s \subseteq u.\end{align*}

Put simply, we compare a candidate system $s \subseteq u$ to all other potential candidate systems $s^{\prime} \subseteq u$, and ensure that its system integrated information is greater than any subset, superset, or paraset of itself (i.e. any overlapping candidate system). Over a universal substrate $u$, non-overlapping complexes are identified recursively (first-maximal complex, then second-maximal complex, and so on).

Finally, it follows from exclusion and the principle of maximal existence that a complex should not only have greater $\varphi _{s}$ than overlapping candidate systems constituted of micro units, but also any overlapping systems constituted of macro units. In the next section, we extend IIT’s mathematical framework to permit evaluation of $\varphi _{s}$ for systems containing macro units.

### Intrinsic units

A complex’s *intrinsic units*—those that account for its experience—maximize its $\varphi _{s}$ while complying with the postulates. It is not a requirement that a complex’s units all share the same grain, so a *system’s grain* refers not to a single grain, but rather to its particular configuration of units at their particular (possibly heterogeneous) grains. A system has a macro grain if it contains at least one macro unit. Evaluating $\varphi _{s}$ for candidate systems at all possible grains requires extending IIT’s mathematical framework as follows.

#### Meso and macro units

Starting from a set of micro units within $U$, a macro unit can be obtained by macroing “over units” (when a macro unit has more than one constituent unit), “over updates” (when a macro unit has more than one update step), or both. Previous work referred to macroing as being “over space” and/or “over time” (Hoel *et al.* 2013; Marshall *et al.* 2018), but we avoid these terms here, because of their metaphysical implications. The IIT framework does not require spacetime to be fundamental.

As mentioned above, micro units are the finest units that can be observed and manipulated, satisfying the minimal requirements for cause-effect power (i.e. physical existence) (Albantakis *et al.* 2023b). These “atoms” of cause-effect power cannot be partitioned into finer constituents, their updates cannot be partitioned into finer updates, and they cannot have more than two states—the minimum necessary to bear a cause and produce an effect—otherwise they would be equivalent to a combination of finer units, contradicting their finest status. Moreover, allowing for an arbitrary number of states would allow for an arbitrary amount of cause-effect power hidden within the unit.

Macro units—from the intrinsic perspective of a complex—are its “units” of cause-effect power and, like micro units, must have a repertoire of exactly two states. Having more than two states would be equivalent to bringing their micro units into play at the macro level, allowing micro mechanisms to distinguish among the macro unit’s states. At the same time, these internal micro-mechanisms would be inaccessible to macro-level partitions. These internal mechanisms would contribute cause-effect power to the macro level that belongs at the micro level, overestimating the amount of causal power that is intrinsic to the macro grain (Supplementary Fig. 1). Of course, from the extrinsic perspective of an experimenter unconcerned with the separation of grains, non-binary macro states are available for observation and manipulation, and can reveal important causal properties of a substrate (see Discussion).

For the purpose of defining units at different grains, we assume that $U = \{U_{1}, U_{2}, \ldots , U_{n}\}$ is a set of micro units. A macro unit $J$ constructed from micro units has four aspects:


(10) \begin{align*}& J = (U^{J}, \tau_{J}, g_{J}, W^{J}),\end{align*}

where $U^{J} \subseteq U$ are its *micro constituents*, $\tau _{J} \in \mathbb{Z}^{+}$ is its *update grain* in terms of micro updates, and $g_{J}$ a mapping from the states of $U^{J}$ over a sequence of $\tau _{J}$ micro updates to the state of $J$:


(11) \begin{align*}& g_{J}: \Omega_{U^{J}}^{\tau_{J}} \rightarrow \{0, 1\}.\end{align*}

The fourth element of a macro unit, $W^{J}$, is its *apportionment* of the complex’s background conditions. For a system at a micro update grain, the current state of its background units provides the context that may partly enable the system’s cause-effect power (Albantakis *et al.* 2023b). At a macro update grain, this context is provided not only by the current state of a system’s background units, but also by the way their state changes over multiple micro updates. This means that background units can potentially mediate cause-effect power among units. However, the cause-effect power of units should not be counted multiple times. Accordingly, two macro units cannot share the same micro constituents, nor can there be overlap among the micro units mediating the effects of different macro units. Background units are thus partitioned into disjoint sets and apportioned to specific macro units in a way that maximizes the complex’s $\varphi _{s}$. $W^{J}$ can be ignored at the micro update grain, because a single micro update does not provide any opportunity for background units to mediate interactions among a system’s units.

**Figure 1.**
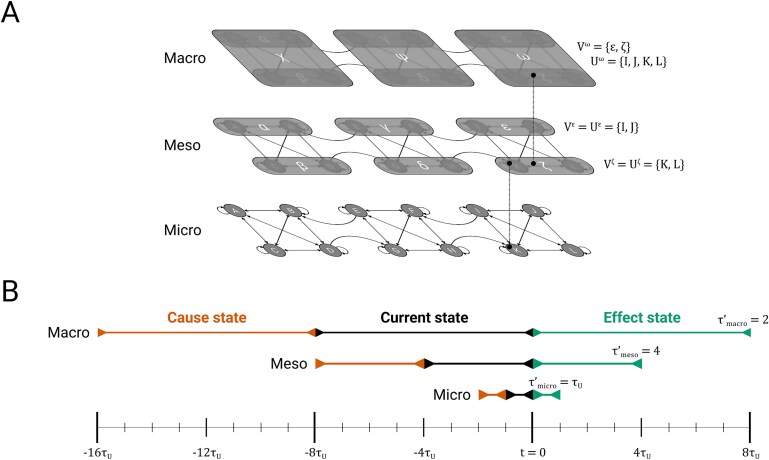
**From micro to macro units.** (A) A universe $U=\{A, B, C, D, E, F, G, H, I, J, K, L\}$, with unspecified transition probability function $\mathcal{T}_{U}$. Although some intrinsic cause-effect power may be associated with units at this micro grain, it is also possible that intrinsic cause-effect power is highest at a macro grain. For example, it might be maximal for the macro system $\{\chi , \psi , \omega \}$, which would mean that this system exists from its own perspective as a system of three macro units. The framework provided in this paper will allow us to assess if this is the case, including whether a macro unit, say $\omega $, can be built using micro constituents $U^\omega =\{I, J, K, L\}$, possibly with intermediate meso constituents $V^\omega =\{\epsilon , \zeta \}$. (B) In addition to defining macro states over groups of units, it is also possible to define macro states over updates of $U$. We depict one hypothetical scenario in which macro units have an update grain equal to 2 meso updates ($\tau ^{\prime}_{\mathrm{macro}}=2$), meso updates have an update grain equal to 4 micro updates ($\tau ^{\prime}_{\mathrm{meso}}=4$), and the micro update ($\tau ^{\prime}_{\mathrm{micro}}= \tau _{U} = 1$) is inherited from $U$. The macro state of a unit is always defined looking back from the current micro instant. Thus, this macro state, while a function of several updates, can change every micro update, in a “sliding window” fashion.

Constructing macro units directly from micro units is a special case of a more general framework. A macro unit may also be built from constituents $V^{J}$ that are themselves macro units at a finer grain—called *meso units*—and the same may be true for the meso units’ constituents, and so forth. That is, a macro unit may be built from one or more levels of meso units sandwiched between it and its constituent micro units $U^{J}$ (Fig. 1). Formally, there is no difference between macro units and meso units, but for clarity, we will hereafter reserve the term “macro” for the grain of an intrinsic unit (when it is not a micro unit), and “meso” for any intermediate grains.

To facilitate the distinction between a unit’s micro constituents $U^{J}$ and its direct constituents $V^{J}$—which may be meso units—we extend our definition of $J$ above to be completely general, covering cases where $J$ is a micro, meso, or macro unit. A unit $J$ has five aspects:


(12) \begin{align*}& J = (U^{J}, V^{J}, \tau^{\prime}_{J}, g^{\prime}_{J}, W^{J}),\end{align*}

where $U^{J} \subseteq U$ are its micro constituents, $V^{J}$ are its constituents (which may be micro or meso units) with current state $v^{J}$ and state space $\Omega _{V^{J}}$, $W^{J}$ is its background apportionment, which must contain the background apportionments of its constituents:


(13) \begin{align*}& W^{V_{i}} \subseteq W^{J} \qquad \forall~ V_{i} \in V^{J},\end{align*}

$\tau ^{\prime}_{J} \in \mathbb{Z}^{+}$ is the update grain over which $J$’s constituents are evaluated to define the state of $J$, and $g^{\prime}_{J}$ is a mapping from the states of $V^{J}$ over a sequence of $\tau ^{\prime}_{J}$ updates of $V^{J}$ to the state of $J$:


(14) \begin{align*}& g^{\prime}_{J}: \Omega_{V^{J}}^{\tau^{\prime}_{J}} \rightarrow \{0, 1\}.\end{align*}

In general, there are $2^\wedge (2^\wedge (\tau ^{\prime}_{J}|V^{J}|)) - 2$ possible mappings from the state of constituents to the state of $J$. It is important to note that when $J$ is constructed from a hierarchy of meso units of increasing grain, the update grain $\tau ^{\prime}_{J}$ and the function $g^{\prime}_{J}$ define a mapping across a *single* level of this hierarchy, from a sequence of states of $V^{J}$ to the state of $J$. If $V^{J}$ is a set of meso units, then $\tau ^{\prime}_{J}$ is the number of *meso* updates that define $J$’s state. There exist additional mappings between $V^{J}$’s constituents and $V^{J}$, and so on, down to the micro constituents $U^{J}$ (with a corresponding nested sequence of background apportionments). Thus, in addition to the update grain of $J$ in terms of its direct constituents ($\tau ^{\prime}_{J}$), this hierarchical sequence of mappings can be used to define an update grain of $J$ in terms of its *micro* constituents, which we label $\tau _{J}$. Similarly, we have a mapping $g_{J}$ from sequences of microstates to the state of $J$:


(15) \begin{align*}& g_{J}: \Omega_{U^{J}}^{\tau_{J}} \rightarrow \{0, 1\}.\end{align*}

For example, in Fig. 1B, $\tau ^{\prime}_{J}=\tau ^{\prime}_{\mathrm{macro}}=2$ and $\tau _{J}=8$, because $J$’s state is defined over a sequence of 2 meso updates, each of which consists of 4 micro updates. Note that unlike $\tau ^{\prime}$, $\tau $ is non-decreasing as a function of the level in the hierarchy. Also note that from the perspective of the system at any given micro instant, the constituents $V^{J}$ of a macro unit are fixed throughout its macro update, though it is possible (through the mapping $g_{J}$) for different micro units to contribute to the state of the macro unit at different micro updates.

#### Applying the postulates to units

A complex, including its units, must comply with IIT’s postulates of physical existence (Albantakis *et al.* 2023b). Like complexes, macro units must have cause-effect power that is intrinsic, specific, irreducible, and definite.

**Figure 2.**
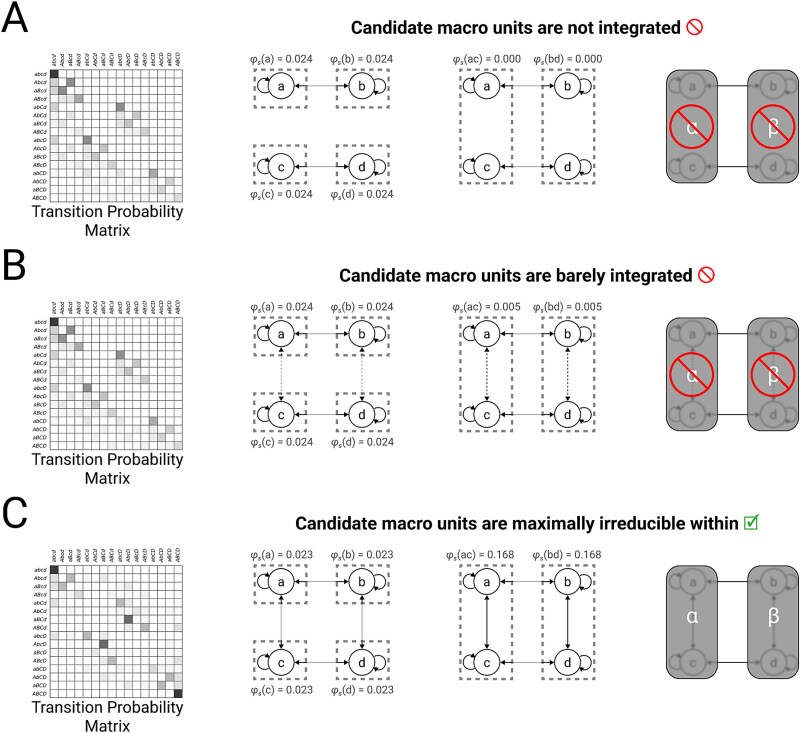
**Out of nothing, nothing comes.** Consider four micro units $\{A, B, C, D\}$ in state $(0, 0, 0, 0)$, constituting universe $U$ with TPM $\mathcal{T}_{U}$. For each micro unit $U_{i}$, when all its inputs are $0$, the probability that its state will be $1$ after the next update is 0.05. This probability is increased by 0.05 if $u_{i}$ itself is currently $1$, and further increased by 0.6 if the state of $U_{i}$’s horizontal neighbor is $1$. (A) Consider the case where there are no connections between vertical neighbors (left). Each micro unit has $\varphi _{s}=0.024$ on its own (middle left), while each pair of vertical neighbors has $\varphi _{s} = 0$ (middle right). Because each pair of vertical neighbors is reducible, they are not valid macro elements (right). (B) Consider the case where vanishingly weak connections are introduced between vertical neighbors, such that the probability that $u_{i}$ will be $1$ after the next update is increased by 0.01 if $U_{i}$’s vertical neighbor is $1$ (left). Although each pair of vertical neighbors is now very weakly integrated with $\varphi _{s} = 0.005$ (middle right), they are not maximally irreducible within (e.g. $\varphi _{s}(a, b) < \varphi _{s}(a)$). The conclusion is the same as for (A): the vertical neighbors are not valid macro elements (right). (C) Finally, consider the case where strong connections are introduced between vertical neighbors, such that the probability that $u_{i}$ will be $1$ after the next update is increased by 0.25 if $U_{i}$’s vertical neighbor is $1$ (left). Integration between vertical neighbors is now sufficiently strong (middle right) that the “maximally irreducible within” criterion is satisfied (middle right vs middle left), so we can consider macro elements built from vertical neighbors (right). There is no guarantee that the macro system consisting of these elements $\{\alpha , \beta \}$ is a complex, but at least we may evaluate that possibility (not shown).

Consider the requirement for integration. Just like a complex, a candidate macro unit that does not satisfy integration, because it is reducible to causally independent subsets of micro units, cannot truly exist as *one* unit. There is nothing unitary about it, except possibly from the extrinsic perspective of an experimenter. Pretending otherwise would be tantamount to building something (a macro unit, and then a complex) out of nothing (non-interacting micro units) (Fig. 2A). As mentioned above, previous versions of the theory (Marshall *et al.* 2018) failed to impose this requirement on macro units directly—only on the system—which required partitions testing system irreducibility to be performed at the micro level, often cutting through macro units. Although this did limit the gerrymandering of macro units and complexes illustrated in Fig. 2, it contradicted the premise that from the intrinsic perspective of the system, it exists as a collection of macro (rather than micro) units.

Consider also the requirement for exclusion. Just as complexes must be definite, so must macro units: there must be a reason why a unit has the border it has. In the case of a complex, that reason is provided by the principle of maximal existence: the border is the one that yields maximal irreducibility. However, unlike complexes, intrinsic units only need to be maximally irreducible *within* (there cannot be any subset with higher integrated information) but not necessarily without (there can be supersets and/or parasets having higher integrated information). If intrinsic units were not required to be maximally irreducible within, one could treat as a macro unit a collection of nearly independent micro units, again building something out of “nearly nothing” (Fig. 2). On the other hand, the units of a complex do not need to be maximally irreducible without, because the irreducibility to be maximized is that of the complex, rather than that of its units. Therefore, the borders of its intrinsic units should be those that maximize the complex’s $\varphi _{s}$, rather than each unit’s $\varphi $. In summary, an intrinsic unit must be a maximally irreducible constituent of a complex (“maximally irreducible within”), rather than a complex itself (“maximally irreducible within and without”).

We now consider the requirements for intrinsic units more formally. To satisfy the intrinsicality, information and integration postulates, a unit $J \in S$ with constituents $V^{J}$ (in current state $v^{J}$) and background apportionment $W^{J}$ must have cause-effect power that is intrinsic, specific, and irreducible:


(16) \begin{align*}& \varphi_{s}(v^{J})> 0.\end{align*}

**Figure 3.**
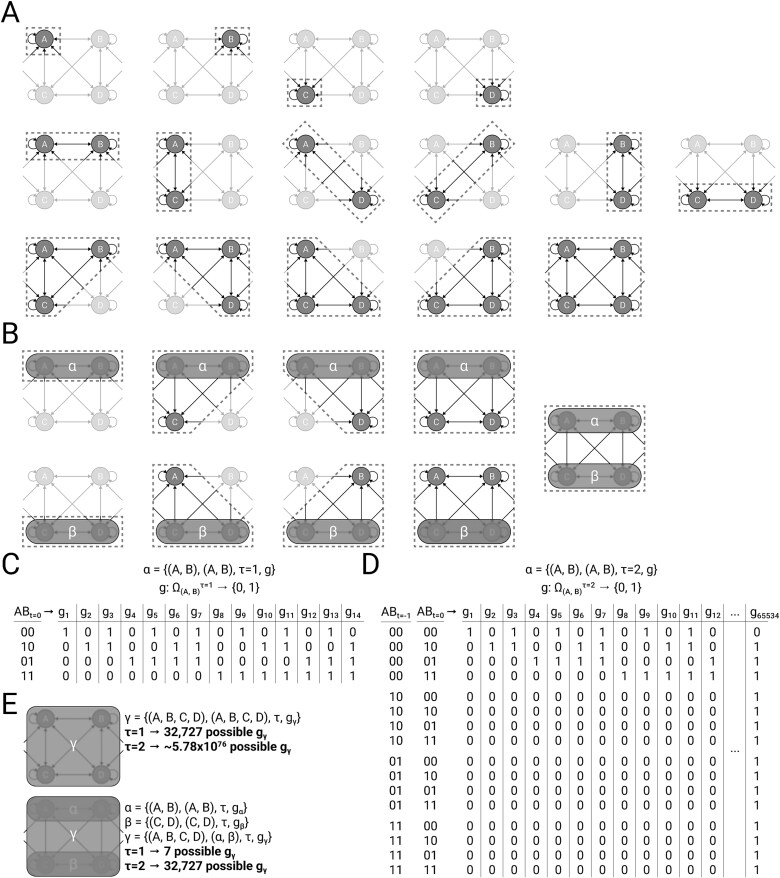
**Defining macro units.** Four micro units $\{A, B, C, D\}$ are embedded within a larger universe $U$, with unspecified transition probability function $\mathcal{T}_{U}$. We wish to know if a macro unit $\gamma $ can be built using micro constituents $U^\gamma =\{A, B, C, D\}$, possibly with intermediate meso constituents $V^\gamma \neq U^\gamma $. (A) To ask if $\gamma =\{(A, B, C, D),...\}$ is admissible as a macro unit, we must first check integrated information $\varphi _{s}$ for every subset of micro units. (B) Suppose we find that $\{A, B\}$ and $\{C, D\}$ are maximally irreducible within (i.e. they satisfy Equation 17). This means that they are potential meso units, labeled $\alpha $ and $\beta $ respectively. To continue verifying that $\gamma =\{(A, B, C, D),...\}$ is admissible as a macro unit, we must now check integrated information for every subset of units that include $\alpha $ and $\beta $ as well. (C) Let S be the macro system containing $\alpha $. For a given candidate unit, say $\alpha =\{(A, B), (A, B), \tau _\alpha =1, g_\alpha )\}$, there are many potential mappings $g_\alpha $ from the states of $V^\alpha =\{A, B\}$ over a sequence of $\tau _\alpha =1$ updates to the state of $\alpha $, but only one (here unspecified) will maximize $\varphi _{s}(s)$. (D) Same as (C), but over a sequence of $\tau _\alpha =2$. Note that it is not only the ultimate state of the micro constituents that determine the macro unit’s state, but the precise sequence of micro states. (E) Depending on which of $\{A, B, C, D\}$ or $\{\alpha , \beta \}$ (or some mixture) is maximally irreducible, $\gamma $’s constituents $V^\gamma $ might be $\{A, B, C, D\}$ or $\{\alpha , \beta \}$ (or some mixture), which in turn will dictate the set of potential mappings from which $g_\gamma $ can be defined, for any given $\tau $. There are far fewer mappings that need to be considered for a macro unit whose constituents are meso units, because the mapping of the macro unit ($\gamma $) is constrained by the mappings of its meso constituents ($\alpha $, $\beta $).

Moreover, to satisfy exclusion, $J$ must have higher integrated information than any other valid system that could be constructed from its micro constituents $U^{J}$ and background apportionment $W^{J}$, at a temporal grain less than or equal to its own:


(17) \begin{align*}& \varphi_{s}(v^{J})> \varphi_{s}(v^{\prime}) \qquad \forall~ V^{\prime} \in f(U^{J}, W^{J}, \tau_{J}),\end{align*}

where $f(U^{J}, W^{J}, \tau _{J})$ is a set function that identifies all valid systems $V^{\prime}$ (ones that satisfy Equations (17] and (19)) whose micro constituents are a subset of $U^{J}$, whose background apportionments are non-overlapping subsets of $W^{J}$, and whose units all have a temporal grain that is less than or equal to $\tau _{J}$. The requirement that intrinsic units be maximally irreducible within applies whether macroing over units (Fig. 3A–C), and/or over updates (Fig. 3D), and applies to units at any grain.

Finally, consider a candidate system:


(18) \begin{align*}& S = \{J_{1}, J_{2}, \ldots, J_{|S|}\}.\end{align*}

If the cause-effect power of a micro unit $U_{i} \in U$ is not to be counted multiple times within $S$, (i) $U_{i}$ cannot be a micro constituent of more than one macro unit; (ii) it cannot be apportioned as background to more than one macro unit; and (iii) it cannot be both a constituent of one macro unit and a mediator for another (this is analogous to the motivation for “screening-off” in other causal inference frameworks (Salmon, 1971; Brandon, 1982; Reichenbach and Reichenbach, 1999; Pearl, 2000)). This puts the following restriction on S:


(19) \begin{align*}& (U^{J} \cup W^{J}) \cap (U^{J^{\prime}} \cup W^{J^{\prime}}) = \varnothing, \qquad \forall~ J, J^{\prime} \in S.\end{align*}

This restriction also ensures that the effects of multiple intrinsic units within a complex are integrated by other intrinsic units and not in the background. Because intrinsic units contribute cause-effect power to the complex but background conditions do not, (i) a complex can use another complex as background, but a unit cannot use another unit as background within a complex; (ii) two complexes can use the same background, but two units cannot use the same background within the same complex.

Having defined the criteria for an admissible system, the definition of a complex can be extended to arbitrary systems of units across grains. Let $\mathbb{P}(u)$ be the set of all valid systems that can be defined from the universe $U$ in state $u$. A system $S = \{J_{1}, \ldots , J_{|S|}\}$ in state $s$ is a complex if it has more integrated information than any other admissible system that overlaps its micro constituents:


(20) \begin{align*}& U^{S} \cap U^{S^{\prime}} \neq \varnothing \Rightarrow \varphi_{s}(s)> \varphi_{s}(s^{\prime}) \qquad \forall ~ s^{\prime} \neq s \in \mathbb{P}(u).\end{align*}

A consequence of this ‘maximally irreducible within’ requirement is that for a given set of micro constituents $U^{J}$, whether an intrinsic unit $J$ is built upon meso units (Fig. 3E, bottom) or it is built directly “in one shot” upon the micro units (Fig. 3E, top) depends on which definition of $V^{J}$ maximizes $\varphi _{s}(v^{J})$. In general, having finer (e.g. micro) constituents means having a larger number of mappings available to $J$ with which to maximize $\varphi _{s}$ at the macro grain, but makes it harder for $V^{J}$ to satisfy the requirement of being “maximally irreducible within.” For finer-grain systems with a large number of constituents, having high $\varphi _{s}$ requires that these units are both highly selective (to support intrinsic information), and have a connectivity structure without fault lines (to support integration) (Marshall *et al.* 2023). By contrast, coarser systems of units (defined from the same microconstituents) have fewer units, each of which can be flexibly defined through intermediate mappings to have the high selectivity and connectivity structure required to support highly integrated information. Thus, although there is no strict requirement that a system of macro units be built up from meso units, there are good reasons to expect that many systems will have this property.

It is worth noting that whether a macro system is built up in levels, which precise macro and meso units it is built from, and which mappings define those units’ state, are all ultimately determined by what maximizes $\varphi _{s}$ at each level of the hierarchy (the level that is maximally irreducible within). Thus, there is always a reason why a complex and its intrinsic units are precisely what they are: the principle of maximal existence.

Also note that the construction of $f(U^{J}, W^{J}, \tau _{J})$ is non-trivial, due to its dependence on $f(U^{S^{\prime}}, W^{S^{\prime}}, \tau _{J})$ for all $S^{\prime}$ with $U^{S^{\prime}} \subseteq U^{J}$; the set of candidate systems depends on the set of admissible macro units, and the set of admissible macro units depends on the set of candidate systems within them (for satisfying “maximally irreducible within”). Practically, the sets need to be derived recursively. The starting point is that each micro unit $U_{i}$ is a potential unit. The set of micro units then defines a set of candidate systems. Those candidate systems are then used as potential meso constituents for defining new potential units, which then leads to new candidate systems. The process can be repeated until convergence, which is guaranteed by the requirement that macro units not overlap.

#### Assessing integrated information of macro systems: a conceptual overview

Having defined intrinsic units and the requirements for a system of intrinsic units to satisfy the postulates, we next outline a general framework for assessing $\varphi _{s}$ that applies to any system, regardless of its units’ grains. In essence, we extend the definition of $\mathcal{T}_{c}$ and $\mathcal{T}_{e}$ to any system, whether it is constituted of micro units or macro units. These TPMs can then be used to compute $\varphi _{s}(s)$ as described in (Albantakis *et al.* 2023b). Here, we describe the process at a high level with some intuition for each step. Then, in the “Assessing integrated information of macro systems: mathematical framework” section, we will introduce some notation and provide a complete mathematical definition of the procedure.

For a system of macro units, we must define the intrinsic cause and effect TPMs ($\mathcal{T}_{c}$ and $\mathcal{T}_{e}$) that describe their cause-effect power within the system, at their defined grains. Intuitively, one might consider using the universe’s micro TPM ($\mathcal{T}_{U}$) to compute conditional probabilities between sequences of micro updates, and then $g_{J}$ to map sequences of micro states to macro states. However, this process can expose micro cause-effect power in the macro TPMs ($\mathcal{T}_{c}$ and $\mathcal{T}_{e}$) that is not intrinsic to the units at their defined grain (i.e. is extrinsic). To discount extrinsic cause-effect power, we employ a four step process (described in “Assessing integrated information of macro systems: mathematical framework”): (i) define modified transition probabilities between micro states; (ii) use these to derive probabilities of sequences of micro updates; (iii) causally marginalize background units; and (iv) map the sequences of micro updates to macro states.

There are two situations in which system TPMs produced without the aforementioned modifications can lead to incorrect conclusions about the intrinsic, integrated cause-effect power of the system. The first is when the cause-effect power of a first macro unit over a third one is mediated by one or more micro constituents of a second macro unit. In this case, the cause-effect power of the these micro constituents will be counted twice: as belonging to both the first and second units. This is avoided by noising indirect pathways among macro units (see Equation 28 below; Supplementary Fig. 2A,B).

The second situation is when a background unit outputs to two different macro units. In this case, the cause-effect power of the same micro unit would be counted twice, as a mediator for both macro units. This can be avoided by noising, for each background micro unit, the inputs from macro units other than the one it is apportioned to [see Equation (29) below; Supplementary Fig. 2A and C].

After discounting the problematic interactions at the micro level, the modified transition probabilities can be used to compute the probability of sequences of microstates for the universe $U$, given a current microstate of the universe. This modified TPM only contains the cause-effect power at the micrograin that maps to cause-effect power at the macro grain. Once the transition probabilities have been extended to sequences, background units are causally marginalized conditional on the current state, resulting in conditionally independent transition probabilities between sequences of micro updates for the micro constituents of the substrate. Finally, the sequences of microstates are mapped to macrostates to create $\mathcal{T}_{c}$ and $\mathcal{T}_{e}$.

The sequencing of these operations is important for the correct treatment of background conditions. First, causally marginalizing the background units happens after the transition probabilities are extended to sequences of micro updates. The conditional causal marginalization ensures that the analysis starts from the current state of background units (the context for the system’s cause-effect power), but does not keep them fixed throughout the analysis. This allows the background conditions to “percolate” and mediate interactions among macro units, rather than being absolutely frozen in the current state. Second, the causal conditioning of background units should occur before sequences of microstates are mapped into macrostates. This is because the background units must be treated at the micrograin when assessing the intrinsic cause-effect power of a system; they are extrinsic to the system, and do not exist as macro units from the intrinsic perspective of the system. While the background units may contribute cause-effect power to macro units within other complexes, it is only their actual state, evaluated at the micro grain, that provides the “background” for analyzing the intrinsic cause-effect power of the system under consideration.

#### Assessing integrated information of macro systems: mathematical framework

When dealing with macro update grains, we require additional notation to accommodate sequences of microstates. Let $u_{t} \in \Omega _{U}$ be the state of $U$ at update $t$. Sequences of micro states are defined using a colon in the subscript; e.g. $u_{(t + 1):(t + \tau )} = (u_{t + 1}, u_{t + 2}, \ldots , u_{t + \tau })$ is the sequence of microstates starting at $t + 1$ and ending at $t + \tau $. We let $t=0$ denote the current micro update, so the current micro state of the universe is $u_{0}$. Negative subscripts ($t < 0$) index the updates that led to the current microstate, and positive subscripts ($t> 0$) index the updates that follow from the current micro state.

The above notation applies to subsets of $U$, as indicated by a superscript. For example,


(21) \begin{align*}& u^{J}_{(-\tau_{J} + 1):0} = \left(u^{J}_{-\tau_{J} + 1}, \ldots, u^{J}_{0}\right)\end{align*}

is the sequence of states of the microconstituents of $J$, starting at $t = -\tau _{J} + 1$ and ending at $t = 0$. Thus, for a macro unit $J$ with macro update grain $\tau _{J}$, its current macro state $j$ depends on the previous $\tau _{J}$ states of its micro constituents $U^{J}$ (Fig. 1B):


(22) \begin{align*}& j = g_{J}\left(u^{J}_{(-\tau_{J} + 1):0}\right).\end{align*}

Consider a system $S = \{J_{1}, \ldots , J_{|S|}\}$ in state $s = (j_{1}, j_{2}, \ldots , j_{|S|})$. Each unit $J_{i}$ (whether micro or macro) has micro constituents $U^{J_{i}} \subset U$, constituents $V^{J_{i}}$, background apportionment $W^{J_{i}} \subseteq W$, an update grain $\tau _{J_{i}} \in \mathbb{Z}^{+}$, and a mapping $g_{J_{i}}: \Omega _{U^{J_{i}}}^{\tau _{J_{i}}} \rightarrow \{0, 1\}$. We denote the system’s micro constituents as:


(23) \begin{align*}& U^{S} = \bigcup_{i = 1}^{|S|} U^{J_{i}},\end{align*}

and its background units as:


(24) \begin{align*}& W = U^{W} = U \setminus U^{S}.\end{align*}

$W$ and $U^{W}$ are equivalent (unlike $U^{S}$ and $S$, for example) and may be used interchangeably where notationally convenient, because background units are always treated at the micro grain. The background apportionments for the system, $W^{S} \subseteq W$, are simply the [non-overlapping, see Equation (19)] apportionments of its constituents:


(25) \begin{align*}& W^{S} = \bigcup_{i = 1}^{|S|} W^{J_{i}} \subseteq W.\end{align*}

After discounting connections extrinsic to the system (see below), the micro units in $W^{J_{i}}$ will receive intact inputs from $U^{J_{i}} \cup W^{J_{i}}$, but not from other units. A consequence is that the background units cannot integrate cause-effect power.

Next, we define generalized cause-and-effect TPMs $\mathcal{T}_{c}$ and $\mathcal{T}_{e}$ for $S$, from which the rest of the framework can be applied as usual. The process proceeds in four steps: (i) starting from the universe TPM $\mathcal{T}_{U}$, for each macro unit $J$, discount any connections that are extrinsic to the system (e.g. cause-effect power from micro grains that does not map to macro grains), yielding modified transition probabilities $\hat{p}_{J}$ between micro states; (ii) extrapolate the modified transition probabilities $\hat p_{J}$ into probabilities for sequences of micro updates given a current micro state; (iii) causally marginalize the background ($W$) conditional on the current sequence of micro states; and (iv) use the mappings $g_{J_{i}}$ to compress the micro state-by-sequence transition probabilities for each macro unit into its macro state-by-state transition probabilities, finally combining these macro transition probabilities for each unit to get macro system TPMs $\mathcal{T}_{c}$ and $\mathcal{T}_{e}$. When no macroing is performed (i.e. all $\tau _{J_{i}} = 1$ and all $g_{J_{i}}$ are identity functions), (i), (ii), and (iv) are trivial, and $\mathcal{T}_{c}$ and $\mathcal{T}_{e}$ work out to be (micro) TPMs as defined in (Albantakis *et al.* 2023b).

**Step 1: discounting connections extrinsic to the system.** We wish to define modified state transition probabilities that reflect the effects of discounting certain micro connections. The specific micro connections to be discounted will depend on the macro unit being updated. For example, if we are updating the state of $J_{1}$ then connections from $J_{2}$ to $J_{1}$ are left intact, but if we are updating the state of $J_{3}$, those connections are noised to prevent $J_{1}$’s micro constituents from mediating other units’ effects. To have connections selectively discounted, we will define a different $\hat p_{J}$ for each macro unit $J \in S$ to be updated, and then combine them later with a product. The product removes any cause-effect power from the TPM whose source is correlations among units due to common input from background units or microconstituents, leaving intact the direct cause-effect power among units.

For a given $J \in S$ and $U_{i} \in U$, we would like to define:


(26) \begin{align*}& \hat p_{J}(u^{\prime}_{i} \mid u) \qquad \begin{matrix} u \in \Omega_{U}, \\ u^{\prime}_{i} \in \{0, 1\}. \end{matrix}\end{align*}

If $U_{i} \in U^{J}$ ($U_{i}$ is a constituent of the to-be-updated macro unit), then no connections are discounted:


(27) \begin{align*}& \hat p_{J}(u^{\prime}_{i} \mid u) = p(u^{\prime}_{i} \mid u), \qquad u \in \Omega_{U}, ~ u^{\prime}_{i} \in \{0, 1\}\end{align*}

If $U_{i} \in U^{S} \setminus U^{J}$ (in the system, but not a constituent of $J$) or $U_{i} \in W \setminus W^{S}$ (a background unit that is not apportioned to any system unit), then all connections should be discounted:


(28) \begin{align*}& \hat p_{J}(u^{\prime}_{i} \mid u) = \frac{1}{|\Omega_{U}|} \sum_{\bar{u} \in \Omega_{U}} p(u^{\prime}_{i} \mid \bar{u}), \qquad u \in \Omega_{U}, ~ u^{\prime}_{i} \in \{0, 1\}.\end{align*}

This ensures that the micro units that constitute macro units do not have a second role as mediators of other macro units’ effects (e.g. $J_{1}$ effects $J_{3}$ through $J_{2}$’s micro constituents).

If $U_{i} \in W^{J_{k}}$ for some $J_{k} \in S$, then all connections from $U^{J_{k}}$ and $W^{J_{k}}$ should be kept intact, but all other connections should be noised (allowing $W^{J_{k}}$ to mediate $J_{k}$’s effects, but no other units’ effects):


(29) \begin{align*}& \hat p_{J}(u^{\prime}_{i} \mid u) = \frac{1}{|\Omega_{U}(u, k)|} \sum_{\bar{u} \in \Omega_{U}(u, k)} p(u^{\prime}_{i} \mid \bar{u}), \qquad u \in \Omega_{U}, ~ u^{\prime}_{i} \in \{0, 1\},\end{align*}

where $\Omega _{U}(u, k) = \{\bar{u} \in \Omega _{U}: \bar{u}^{W^{J_{k}}} \cup \bar{u}^{J_{k}} \subset u\}$ is the set of all universe states where the state of $J_{k}$’s micro constituents ($u^{J_{k}}$) and background apportionment ($w^{J_{k}} = u^{W^{J_{k}}}$) are consistent with $u$. Averaging over system states discounts (noises) all micro connections to $W^{J_{k}}$ from outside $U^{J_{k}} \cup W^{J_{k}}$.

The modified unit probabilities, $\hat p_{J}(u^{\prime}_{i} \mid u)$, can then be combined to create a modified universe TPM that contains only the connections required to update the state of $J$,


(30) \begin{align*}& \hat p_{J}(u^{\prime} \mid u) = \prod_{i = 1}^{n} \hat p_{J}(u^{\prime}_{i} \mid u), \qquad u, u^{\prime} \in \Omega_{U}.\end{align*}

For an illustrated example, see Supplementary Fig. 2.

**Step 2: obtaining probabilities for sequences of micro updates.** The modified transition probabilities can now be used to compute the probability of any sequence of $\tau _{J}$ micro states $u_{(t + 1):(t + \tau _{J})}$, conditional on any given state $u_{t}$:


(31) \begin{align*}& \hat p_{J}(u_{(t + 1):(t + \tau_{J})} \mid u_{t}) = \prod_{i = 1}^{\tau_{J}} \hat{p}_{J}(u_{t + i} \mid u_{t + i - 1}) \qquad \begin{matrix} u_{t} \in \Omega_{U}, \\ u_{(t+1):(t+\tau_{J})} \in \Omega_{U}^{\tau_{J}}. \end{matrix}\end{align*}

Note that $\hat p_{J}$ may denote state-to-state transition probabilities [as in Equation (30), or the right side of Equation (31)] or state-to-sequence transition probabilities [as in the left side of Equation (31)], depending on its arguments (e.g. $\hat p_{J}(u_{t + 1} \mid u_{t})$ vs. $\hat p_{J}(u_{(t + 1):(t + \tau _{J})} \mid u_{t})$).

**Step 3: causally marginalizing the background.** For each unit’s modified transition probability function $\hat p_{J}$, cause and effect versions $\hat p_{J}^{c}$ and $\hat p_{J}^{e}$ are computed by causally marginalizing the background units conditional on the sequence of $\tau _{J}$ micro updates ending at the current micro update, $u_{(-\tau _{J} + 1):0}$. This entails taking a weighted average of state-to-sequence transition probabilities over potential background states, weighting each by the probability $q_{c/e}(w \mid u_{(-\tau _{J} + 1):0})$ of that background state given the previous $\tau _{J}$ micro states of the universe [analogous to Equations 3-4]:


(32) \begin{align*} \hat p_{J}^{c/e}\left(u^{S}_{(t + 1):(t + \tau_{J})} \mid u_{t}^{S}\right) = \ & \sum_{w \in \Omega_{W}} q_{c/e}(w \mid u_{(-\tau_{J} + 1):0}) \hat p_{J}\left(u^{S}_{(t + 1):(t + \tau_{J})} \mid u_{t}^{S}, w\right) \nonumber \\[-6pt] &\times \begin{matrix} u_{t}^{S} \in \Omega_{U^{S}}, \\ u^{S}_{(t + 1):(t + \tau_{J})} \in \Omega_{U^{S}}^{\tau_{J}}. \end{matrix}\end{align*}

For evaluating effects of the current state, the relevant state is the current state of the background units, $w_{0}$, which is fully determined by the current state of the universe:


(33) \begin{align*}& q_{e}(w \mid u_{(-\tau_{J} + 1):0}) = \begin{cases} 1 \text{ if} \ w = u_{0} \setminus u^{S}_{0} \\ 0 \text{ otherwise,} \end{cases} \qquad w \in \Omega_{W}, ~ u_{(-\tau_{J} + 1):0} \in \Omega_{U}^{\tau_{J}}.\end{align*}

For evaluating causes, because of the Markov property, the only relevant background state is $w_{-\tau _{J}}$. $\mathcal{T}_{U}$ is used in combination with Bayes’ rule to determine a probability distribution for $u_{-\tau _{J}}$, conditional on the sequence of micro states $u_{(-\tau + 1):0}$ Again, due to the Markov property, only the earliest micro state in the sequence (i.e. only $u_{-\tau _{J} + 1}$) is required for the Bayesian computation. A uniform marginal distribution of the previous updates is assumed (i.e. maximum uncertainty about prior states, see also “Cause-effect power at the micro grain”):


(34) \begin{align*}& q_{c}(w \mid u_{(-\tau_{J} - 1):0}) \!=\! \frac{\sum\limits_{\bar{u}^{S} \in \Omega_{U^{S}}} p(u_{-\tau_{J} + 1} \!\mid w, \bar{u}^{S})}{\sum\limits_{\bar{u}} p(u_{-\tau_{J} + 1}\!\mid \bar{u})} \quad w \in \Omega_{W}, ~ u_{(-\tau_{J} + 1):0} \in \Omega_{U}^{\tau_{J}}.\end{align*}

**Step 4: compressing state-by-sequence transition probabilities into macro-state TPMs.** Finally, micro state-to-sequence transition probabilities are mapped to probabilities of individual macro updates. First, for each macro unit, we obtain the probability of transitioning to each of its macro states, given each possible micro state of the system:


(35) \begin{align*}& \hat p_{J}^{c/e}\left(j \mid u_{t}^{S}\right) = \sum_{\bar{u}^{S}_{(t + 1):(t + \tau_{J})} \in D_{J}(j)} \hat p_{J}^{c/e}\left(\bar{u}^{S}_{(t + 1):(t + \tau_{J})} \mid u_{t}^{S}\right) \qquad \begin{matrix} u_{t}^{S} \in \Omega_{U}, \\ j \in \{0, 1\}, \end{matrix}\end{align*}

where $D_{J}(j)$ is the set of sequences of micro states that are mapped to $J = j$:


(36) \begin{align*}& D_{J}(j) = \left\{u_{(t + 1):(t + \tau_{J})}^{S} \in \Omega_{U^{S}}^{\tau_{J}}: g_{J}\left(u^{J}_{(t + 1):(t + \tau_{J})}\right) = j \right\}.\end{align*}

Then we map each current microsystem state $u^{S}$ to the corresponding macrostate. Generally, each current microstate will be mapped to different current macrostates some proportion of the time, depending on the sequence of microstates that led to it, and on the mapping $g_{J}$. For each macro system state $s$:


(37) \begin{align*}& \hat p_{J}^{c/e}(j \mid s) = \sum_{\bar{u}^{S} \in \Omega_{U^{S}}} r(\bar{u}^{S}, s)\hat p^{c/e}_{J}(j \mid \bar{u}^{S}),\end{align*}

where $r(u^{S}, s)$ is the proportion of sequences of $\tau _{J}$ microstates (e.g. $u_{(-\tau _{J} + 1):0}$) that end with $u^{S}$ (i.e. $u^{S}_{0} = u^{S}$), among all the sequences of micro states that get mapped to system state $s$:


(38) \begin{gather*} r(u^{S}, s) = \frac{\left|\left\{u^{S}_{(-\tau_{J} + 1):0} \in D_{S}(s): u^{S}_{0} = u^{S} \right\}\right|}{|D_{S}(s)|}, \qquad u^{S} \in \Omega_{U^{S}}, ~ s \in \Omega_{S}, \end{gather*}

(39) \begin{align*} D_{S}(s) =& \left\{u^{S}_{(-\tau_{J} + 1):0} \in \Omega_{U^{S}}^{\tau_{J}}: \left(g_{1}(u^{J_{1}}_{(-\tau_{J} + 1):0}\right), \ldots, g_{|S|}\left(u^{J_{|S|}}_{(-\tau_{J} + 1):0})\right) = s\right\}, \nonumber \\[-6pt] & \qquad\qquad\qquad\qquad\qquad\qquad\qquad\qquad\qquad\qquad\quad s \in \Omega_{S}. \end{align*}

Operationally, this amounts to a procedure where perturbing a macro unit into its state is achieved by perturbing its micro constituents into all possible microstate sequences that map to the corresponding macro state, with equal probability.

The probability functions $\hat p^{c/e}(j \mid s)$ define the probability of the future macro state of $J$ given the current macro state of $S$. Finally, we combine the functions for each $J$ as a product (establishing conditional independence and removing any correlations due to extrinsic factors):


(40) \begin{align*}& \hat p^{c/e}_{S}(s^{\prime} \mid s) = \prod_{j^{\prime} \in s^{\prime}} \hat p_{J}^{c/e}(j^{\prime} \mid s), \qquad s, s^{\prime} \in \Omega_{S}\end{align*}

where $s^{\prime} = (j^{\prime}_{1}, \ldots , j^{\prime}_{|S|})$.

For a system of macro units, its $\varphi _{s}$ value is computed from the cause and effect TPMs of $S$:


(41) \begin{align*} \mathcal{T}_{c} &\equiv \hat p_{S}^{c}(s^{\prime} \mid s) \qquad s, s^{\prime} \in \Omega_{S}, \end{align*}

(42) \begin{align*} \mathcal{T}_{e} &\equiv \hat p_{S}^{e}(s^{\prime} \mid s) \qquad s, s^{\prime} \in \Omega_{S}, \end{align*}

as described in (Albantakis *et al.* 2023b). It is worth noting that once $\mathcal{T}_{c}$ and $\mathcal{T}_{e}$ have been derived, there is no further reference to the background units, the grain of the units, or their micro constituents. For example, to assess the intrinsic information of the system, the macro units are perturbed, equally likely, into all possible states, regardless of whether or not this corresponds to a uniform distribution for the states of the micro constituents. The TPMs are taken to describe the intrinsic cause-effect power of the system’s units, at their particular grain.

## Examples

In this section, the framework is applied to three example systems. The examples demonstrate that intrinsic cause-effect power can be higher for a system of macro units than for any system of the corresponding micro units, extending results from earlier work (Hoel *et al.* 2016; Marshall *et al.* 2018) to the updated framework (Albantakis *et al.* 2023b). Computations of integrated information were performed using PyPhi (Mayner *et al.* 2018). In what follows, we omit the state as input to $\varphi _{s}$ (e.g. $\varphi _{s}(\{A, B\})$) when the state of the units can be inferred from the context of the example.

To have intrinsic cause-effect power, a system should self-define a repertoire of possible alternative states. Accordingly, throughout the examples, we consider TPMs (and thus units) with some amount of indeterminism, ensuring that there is always a repertoire of potential states. This notion of ‘intrinsic differentiation’ will be further developed in forthcoming work.

### Example 1: a minimal macro complex

The minimal system whose $\varphi _{s}$ peaks at a macro grain is constituted of two micro units and one micro update, because a single micro unit’s $\varphi _{s}$ can never peak at a macro grain. Due to the Markov property, considering a single unit over a macro update grain will compound the indeterminism from its micro updates, reducing its intrinsic information, and therefore its $\varphi _{s}$. Moreover, because it is already a single unit, there is no potential for the macro system to be more integrated than the corresponding micro system.

**Figure 4.**
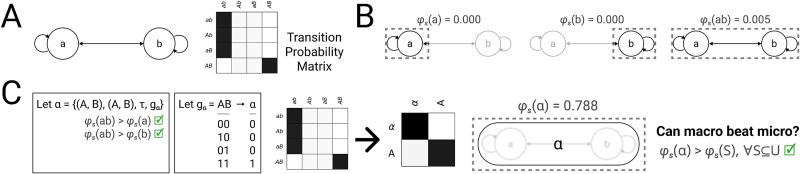
**A minimal macro complex.** (A) Consider two micro units $\{A, B\}$ in state $(0,0)$, with TPM $\mathcal{T}_{U}$. For illustrative purposes, capitalization denotes the state of each unit, both in causal network diagrams and TPM state labels (e.g. state $(0,1)$ is written $aB$). (B) System integrated information $\varphi _{s}(s)$ must be checked for each subset of micro units $S \in \mathbb{P}(\{A, B\})$. Greyed-out units are background. Notice that $\{A, B\}$ is maximally irreducible within, with $\varphi _{s}(\{A,B\}) = 0.005$, greater than either $\varphi _{s}(\{A\})=0$ or $\varphi _{s}(\{B\})=0$. (C) Since $\{A, B\}$ is maximally irreducible within, we may consider a potential macro unit, labeled $\alpha $. The mapping $g_\alpha $ maximizes $\varphi _{s}(\alpha )$, resulting in macro TPM $\mathcal{T}^{S}$. This candidate system $\{\alpha \}$ in state $(0)$ (given by $g_\alpha $) has system integrated information $\varphi _{s}=0.788$, greater than any of the micro level candidate systems in (B).

Consider two micro units $\{A, B\}$ in state $(0,0)$, constituting universe $U$ with TPM $\mathcal{T}_{U}$ (Fig. 4A). Each micro unit $U_{i}$ has the same function: When $u_{i}$ and its neighbor are both $0$, the probability that $U_{i}$’s state will be $1$ after the next update is $0.05$. If $u_{i}$ is $1$ and its neighbor is $0$, this probability is unaffected. However, if $u_{i}$ is $0$ and its neighbor is $1$, this probability is increased to $0.06$. Finally, if *both*  $u_{i}$ and its neighbor are currently $1$, the probability that $u_{i}$ will be $1$ after the next update is increased to $0.95$. Thus, each micro unit approximates a noisy logical AND function over itself and its neighbor, with a weak independent influence from its neighbor when $u_{i}$ is $0$.

We first assess the system integrated information $\varphi _{s}$ of all possible candidate systems of micro units (Fig. 4B). At the micro level, the system {A, B} is maximally irreducible within, because the system integrated information of $\{A, B\}$ ($\varphi _{s} = 0.005$) is greater than for $\{A\}$ or $\{B\}$ individually ($\varphi _{s} = 0$). Because $\{A, B\}$ is maximally irreducible within, it can be considered as the constituents of a macro unit $\alpha $. There are 14 possible mappings for the state of $\alpha $ (Fig. 3C). Testing all possible mappings with $\tau = 1$, we find that the mapping shown in Fig. 4C maximizes the system integrated information of $\{\alpha \}$. Under the mapping shown in Fig. 4C, $\alpha $ behaves as a noisy COPY unit; when it is in state $0$, it is likely to stay in state $0$, while when it is in state $1$, it is likely to stay in state $1$. For the mapping shown in Fig. 4C, the macro system has $\varphi _{s} = 0.788$, which is greater than any system at the micro grain. Thus, this is the minimal example of system integrated information peaking at a macro grain.

### Example 2: coarse-graining

**Figure 5.**
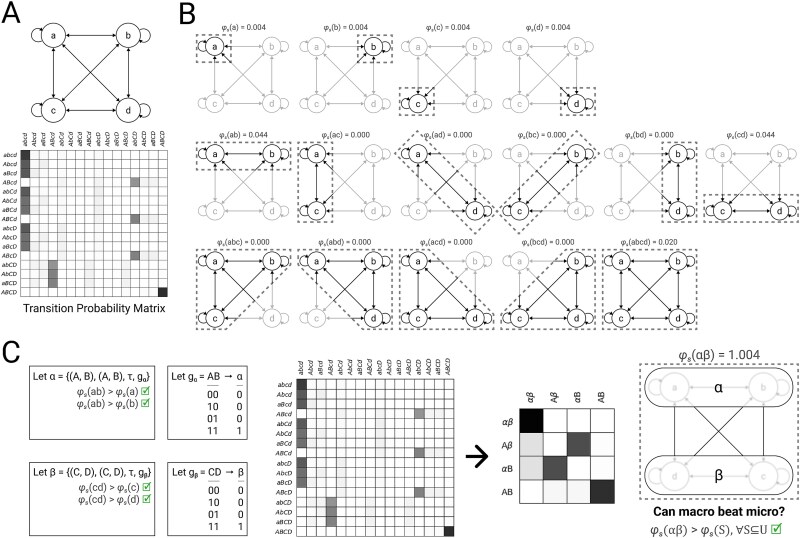
**Coarse-graining.** (A) Consider four micro units $\{A, B, C, D\}$ in state $(0,0,0,0)$, with TPM $\mathcal{T}_{U}$. For illustrative purposes, capitalization denotes the state of each unit, both in causal network diagrams and TPM state labels (e.g. state $(0,1,0,0)$ is written $aBcd$). (B) System integrated information $\varphi _{s}(s)$ must be checked for each subset of micro units $S \in \mathbb{P}(\{A, B, C, D\})$. Greyed-out units are background. Notice that $\{A, B\}$ and $\{C, D\}$ are maximally irreducible within. In the case of $\{A, B\}$: $\varphi _{s}(\{A,B\}) = 0.044$, greater than either $\varphi _{s}({A})=0.004$ or $\varphi _{s}(\{B\})=0.004$. (C) Since $\{A, B\}$ and $\{C, D\}$ are maximally irreducible within, we may consider their potential macro units, labeled $\alpha $ and $\beta $ respectively. One possible pair of mappings for these macro units are $g_\alpha $ and $g_\beta $, resulting in macro TPM $\mathcal{T}^{S}$. This candidate system $\{\alpha , \beta \}$ in state $(0, 0)$ (given by $g_\alpha $, $g_\beta $) has system integrated information $\varphi _{s}=1.004$, greater than any of the micro level candidate systems in (B). Thus, although we would have to check all other valid macro unit definitions and mappings in order to determine whether this macro system is *maximally* irreducible relative to all others, we can conclude that intrinsic cause-effect power will be higher at a macro level than at the micro level—we know that we can do at least as well as $\varphi _{s}=1.004$.

Consider four micro units $\{A, B, C, D\}$ in state $(0,0,0,0)$, constituting universe $U$ with TPM $\mathcal{T}_{U}$ (Fig. 5A). Each micro unit $U_{i}$ has the same function: when all its inputs are $0$, the probability that its state will be $1$ after the next update is 0.05. This probability is increased by 0.01 if $u_{i}$ itself is currently $1$. Thus, there is a very weak tendency for a unit that is $1$ to remain $1$. The probability that $u_{i}$ will be $1$ after the next update is increased by 0.1 if the state of $U_{i}$’s horizontal neighbor is $1$. For example, $A$ is more likely to be $1$ after the next update if $B$ is currently $1$. Finally, the probability that $u_{i}$ will be $1$ after the next update is increased by 0.8 if *both* its vertical neighbor and its diagonal neighbor are currently $1$. For example, $A$ is very likely to be $1$ after the next update if both $C$ and $D$ are currently $1$. Thus, each micro unit approximates a noisy logical AND function over its vertical and diagonal neighbors, with a weak independent influence from its horizontal neighbor, and very weak self-influence. Because each unit’s future state is mostly dictated by its vertical and diagonal neighbors (e.g. $A$’s future state depends most heavily on the current states of $C$ and $D$), and because horizontal neighbors share the same vertical and diagonal neighbors, (e.g. both $A$ and $B$ are dominated by $C$ and $D$), we expect that macroing horizontal neighbors into macro units will reduce the indeterminism and degeneracy associated with the micro units (by combining multiple low probability micro states into a single higher probability macro state), and thereby increase cause-effect power (Hoel *et al.* 2013; 2016; Marshall *et al.* 2018).

To confirm this intuition, we first assess the system integrated information $\varphi _{s}$ of all possible candidate systems of micro units (Fig. 5B). At this micro level, the two candidate systems with the most irreducible cause-effect power are $\{A, B\}$ and $\{C, D\}$, both with $\varphi _{s} = 0.044$. Because these candidate systems are maximally irreducible within (i.e. $\varphi _{s}(\{A,B\})> \varphi _{s}(S) \quad \forall S \subset \{A, B\}$), they satisfy Equation (17) and can be considered as macro units. Notice that although $\{A, B, C, D\}$ as a whole has irreducible cause-effect power ($\varphi _{s} = 0.020$), it is not maximally irreducible within (e.g. $\varphi _{s}(\{A, B\})> \varphi _{s}(\{A, B, C, D\})$) and *cannot* be considered as a macro unit.

Let macro unit $\alpha $ be defined from micro constituents $\{A, B\}$, and $\beta $ from $\{C, D\}$. There are 14 possible mappings for each macro unit (Fig. 3C). In particular, the mapping shown in Fig. 5C seems promising, because it ought to decrease both the indeterminism and the degeneracy that are present in the microsystem. This class of mapping, in which the state of the macro unit is a simple function of the number of constituents in state $1$, has also been referred to as “coarse-graining” (Hoel *et al.* 2013; 2016). Coarse-graining corresponds to the typical notion of a macro state in statistical physics (Marshall *et al.* 2018). Under the mapping shown in Fig. 5C, each macro unit’s state is $1$ if and only if both its micro constituents are $1$. When one macro unit is $1$, odds are that the other macro unit will be $1$ after the next update. When one macro unit is $0$, odds are that the other macro unit will be $0$ after the next update. Thus, the macro system behaves something like two reciprocally connected COPY gates, with some additional complexity provided by the connections between horizontal neighbors at the micro level. This is reflected in the macro system’s TPM (Fig. 5C, middle). Indeed, when we measure the system integrated information of $\{\alpha , \beta \}$, we find $\varphi _{s}(\{\alpha , \beta \}) = 1.004$, demonstrating that this system of macro units has more irreducible, intrinsic cause-effect power than any candidate system built without macro units (Fig. 5C, right).

### Example 3: black-boxing

**Figure 6.**
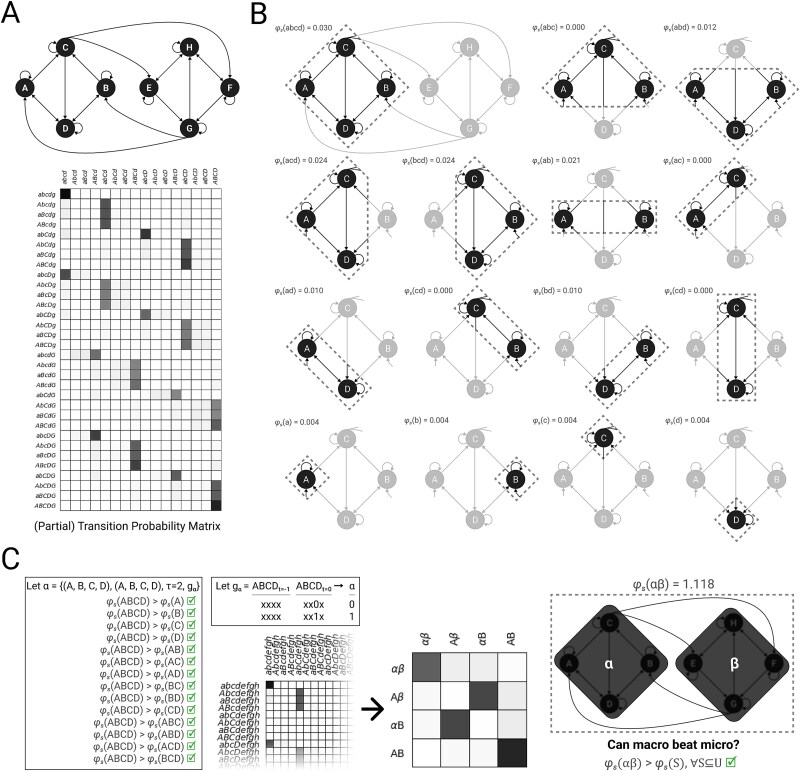
**Black-boxing.** (A) Consider eight micro units $\{A, B, C, D, E, F, G, H\}$ in state $(1, 1, 1, 1, 1, 1, 1, 1)$, with TPM $\mathcal{T}_{U}$. Because of space limitations in this and subsequent panels, we illustrate some analysis steps for the left half of the system only (i.e. $\{A, B, C, D\}$), but all calculations were done using the full eight-unit system. For example, although the full TPM is used for all calculations, a partial TPM illustrating the behavior of $\{A, B, C, D\}$ is shown here. Rows are past system states and columns are future states. (B) System integrated information $\varphi _{s}(s)$ must be checked for each subset of micro units $S \in \mathbb{P}(\{A, B, C, D, E, F, G, H\})$. Here, because of space limitations, we illustrate checks for $S \in \mathbb{P}(\{A, B, C, D\})$. Notice that $\{A, B, C, D\}$ is maximally irreducible within. (C) Since $\{A, B, C, D\}$ is maximally irreducible within, we may consider its potential macro unit, labeled $\alpha $. One possible mapping for $\alpha $ with $\tau = 2$ is shown. Since the full system is symmetric, $\{E, F, G, H\}$ can be considered as a potential macro unit $\beta $ with analogous $g_\beta $, resulting in macro TPM $\mathcal{T}^{S}$. This candidate system $\{\alpha , \beta \}$ in in state $(1, 1)$ (given by $g_\alpha $, $g_\beta $) has system integrated information $\varphi _{s}=1.118$, greater than all of the micro level candidate systems ($\max \varphi _{s} = 0.135$, not shown, but see (B) for a subset). Thus, although we would have to check all other valid macro unit definitions and mappings in order to determine whether this macro system is *maximally* irreducible relative to all others, we can conclude that intrinsic cause-effect power will be higher at a macro level than at the micro level—we know that we can do at least as well as $\varphi _{s}=1.118$.

Consider eight micro units $\{A, B, C, D, E, F, G, H\}$ in state $(1, 1, 1, 1, 1, 1, 1, 1)$, constituting universe $U$ with TPM $\mathcal{T}_{U}$ (Fig. 6A). The left half of the system and the right half of the system ($\{A, B, C, D\}$ and $\{E, F, G, H\}$, respectively) are mirror images of each other, so for simplicity consider the left half. For every unit $U_{i}$, the probability that its state will be $1$ after the next update is marginally higher if its current state is $1$. $C$ approximates a noisy logical OR function of $A$ and $B$, which in turn approximate a noisy logical COPY function of $C$’s image $G$. When $A$, $B$, or $C$ are $1$, the probability that $D$ is $1$ after the next update increases linearly. $D$’s current state also has weak influence on the future state of $A$ and $B$. Roughly speaking, we can think of the two halves of the system as copying each other’s state, but whereas a disruption to any of the connections *within* either half will moderately disrupt this function, a disruption to any of the connections *between* halves will severely disrupt it. It is reasonable to expect that macroing the left half of the system and the right half of the system into separate macro units, and treating the macro units’ states as a simple function of $C$ and $G$’s states, will increase intrinsic cause-effect power. This class of mapping, in which the state of the macro unit is determined only by the state of specific constituents, ignoring others, has also been referred to as “black-boxing.” Black boxes correspond to the typical notion of macro units in the special sciences, because they are constituted of heterogeneous micro units that are often compartmentalized and have highly specific functions, which would be muddled by averaging (Marshall *et al.* 2018).

To compare the cause-effect power of the micro and macro systems, we first assess the system integrated information $\varphi _{s}$ of all possible candidate systems of micro units (Fig. 6B). Note that, in addition to the candidate systems shown in Fig. 6B, all candidate systems of five units (e.g. $\{A, B, C, D, E\}$), six units (e.g. $\{A, B, C, D, E, F\}$), and seven units (e.g. $\{A, B, C, D, E, F, G\}$) were evaluated (not shown). At the micrograin, the maximum value of $\varphi _{s}$ is $0.135$ ($S = \{A, C, E, G\}$ and symmetric systems). At the micrograin, the two candidate systems that we hypothesized would make good macro units ($\{A, B, C, D\}$ and $\{E, F, G, H\}$) are maximally irreducible within, with $\varphi _{s} = 0.030$. Because these candidate systems are maximally irreducible within (i.e. $\varphi _{s}(\{A,B,C,D\})> \varphi _{s}(S) \quad \forall S \subset \{A,B,C,D\}$), they satisfy condition (17) and can be considered as macro units.

Let macro unit $\alpha $ be defined from micro constituents $\{A, B, C, D\}$, and $\beta $ from $\{E, F, G, H\}$. Our mapping of interest, where the state of $\alpha $ is dictated by the state of its output unit $C$ over two micro updates ($\tau = 2$), is shown in Fig. 6C. Under this mapping, the macro system behaves something like two reciprocally connected COPY gates, with some additional complexity provided by the connections within each macro unit. This is reflected in the macro system’s TPM (Fig. 6C, middle), which is very similar to the macro TPM obtained in the previous example (Fig. 5C, middle). Indeed, when we measure the system integrated information of $\{\alpha , \beta \}$, we find $\varphi _{s}(\{\alpha , \beta \}) = 1.118$, demonstrating that this system of macro units has more irreducible, intrinsic cause-effect power than any candidate system built without macro units (Fig. 6C, right).

## Discussion

This work presents a framework for identifying the grain of a system’s units in a way that is consistent with IIT’s postulates and principles. To this end, we introduced the notion of a substrate’s intrinsic units—those that account for its experience, whatever their grain may be—and formalized what it means for these units, as part of a substrate of consciousness, to satisfy IIT’s postulates of physical existence. Based on this framework, we demonstrated that macro grain systems (systems constituted of one or more macro units) can have more irreducible, intrinsic cause-effect power—as measured by system integrated information ($\varphi _{s}$)—than the corresponding micro grain systems.

IIT’s existence postulate requires that macro units have cause-effect power (that they “take and make a difference”), as established operationally by manipulating and observing their state. The intrinsicality, information, integration, and exclusion postulates require that the cause-effect power of macro units be intrinsic, specific, irreducible ($\varphi _{s}> 0$), and definite in grain. Based on IIT’s principle of maximal existence (among competing existents, the one that actually exists is the one that exists the most), the grain is such that (i) each unit is maximally irreducible “within” (it has greater $\varphi _{s}$ than any combination of its constituents) and (ii) the units taken together maximize irreducibility of $\varphi _{s}$ over their substrate. These then constitute the complex’s “intrinsic units.” From the perspective of the complex they constitute, intrinsic units have no internal structure of their own and exist in one of two alternative macro states.

Searching across grains for maxima of $\varphi _{s}$ only makes sense if cause-effect power can peak at macro grains, a phenomenon known as *causal emergence* (Yuan *et al.* 2024). Although IIT is not an emergentist theory (Chis-Ciure 2024b), its framework for identifying intrinsic existence at macro grains resembles a framework for causal emergence, and the first rigorous, quantitative theory of causal emergence (Hoel *et al.* 2013) was motivated in part by the need to identify intrinsic existence at macro grains (Hoel *et al.* 2016; Marshall *et al.* 2018). IIT has also inspired or influenced other recent causal emergence frameworks (Mediano, 2019; Rosas *et al.* 2020; Grasso *et al.* 2021; Sampson, 2024). For a detailed comparison of IIT with several frameworks for quantifying emergence, including causal emergence, see the supplementary material. It is important to keep in mind that differences between frameworks follow from differences in purpose and in underlying model assumptions. Whereas IIT is primarily concerned with consciousness (intrinsic existence) and the identification of intrinsic, irreducible cause-effect power as dictated by the postulates, other frameworks will have other objectives. For example, their interests may be in identifying macroscale descriptions of systems that improve prediction (Crutchfield, 1994; Shalizi and Moore, 2003; Rosas *et al.* 2020) or explanation (Marrow *et al.* 2020; Klein *et al.* 2021; Zhang and Liu, 2022), achieving dimensionality reduction or descriptive compression while preserving underlying microscale dynamics (Klein and Hoel, 2020; Rosas, 2024; Zhang *et al.* 2025), or finding closed levels of description (Chang *et al.* 2020; Rosas, 2024).

The examples presented in this work demonstrate that IIT 4.0’s measure of integrated information ($\varphi _{s}$), calculated on the basis of intrinsic units, can indeed peak at macro grains. We also show that a macro system can have greater integrated information than the corresponding micro system if it is associated with reduced indeterminism and degeneracy of state transitions, such that the selectivity of intrinsic causes and effects is correspondingly increased. In IIT 4.0, this increased selectivity is captured naturally by $\varphi _{s}$ because of its formulation in terms of intrinsic information (Barbosa *et al.* 2020; 2021; Marshall *et al.* 2023). Moreover, a system of macro units can have greater integrated information than the corresponding micro units if integration is higher at the macro level. These properties of $\varphi _{s}$ are consistent with other measures of causation (Comolatti *et al.* 2025), including previous measures of integrated information (Hoel *et al.* 2016; Marshall *et al.* 2018).

To achieve a high value of $\varphi _{s}$, systems of any grain must balance integration with differentiation. Whether $\varphi _{s}$ will increase with a larger number of units depends on a balance between how much additional cause and effect information the system can specify (because its state repertoire has expanded), how much the selectivity of causes and effects within the system is reduced (because cause and effect information is spread over additional states, even more so if the additional units bring increased noise), and how well integrated the additional units are with the rest of the system (Barbosa *et al.* 2020; 2021; Marshall *et al.* 2023). Thus, a system of many units can only “hang together well” as an intrinsic entity if its units are themselves highly integrated and are appropriately interconnected, say as a dense, directed lattice (Albantakis *et al.* 2023b). We conjecture that macro units built upon a hierarchy of meso units may play a crucial role in allowing large systems to exist as maxima of intrinsic, irreducible cause-effect power. Hierarchies of this sort appear to be a common feature of biological systems, and their presence may be related to intuitive notions of complexity (Marchese *et al.* 2022; Hoel, 2025).

In general, macro grains with $\varphi _{s}$ values higher than *most* finer or coarser grains—that is, local or “extrinsic” maxima of integration and causal efficacy (Hoel *et al.* 2013)—are likely to capture relevant levels of substrate organization by “carving nature at its joints” (Hamilton and Cairns, 1963). In the brain, for example, these might correspond to proteins, ion channels, organelles, synaptic vesicles, synapses, neurons, groups of tightly interconnected neurons, and so on. Such “extrinsic units,” well-suited to manipulations and observations by neuroscientists, are critical for understanding how the system works. In fact, IIT’s toolbox can in principle be employed to rigorously characterize such extrinsic units. However, according to IIT, there is a critical difference between these locally maximal grains and the absolute maximal grain whose “intrinsic units” maximize $\varphi _{s}$ within and without: only the latter constitutes the substrate of consciousness and accounts for the way the experience feels—all other levels of organization do not exist from the intrinsic perspective.

The exclusion of non-maximal substrates—including units of non-maximal grain—is demanded by IIT’s exclusion postulate and can explain (i) why systems at certain scales (e.g. individual brains) are conscious while others (e.g. nations of people) are not, and (ii) why we are conscious of some contents (e.g. motion in a film presented at 30 frames/second) and not others (e.g. of individual neurons firing individual spikes). For example, assuming the grain of intrinsic units in the brain is that of minicolumns, the update grain might be on the order of 30 ms (in line with estimates of the duration below which non-simultaneous sensory stimuli are perceived as being simultaneous, or changing stimuli are perceived as static (White, 2018)). From the extrinsic perspective of an experimenter, several update grains may be critical to understand different kinds of causal interactions—finer grains for events such as ion channel opening, quantal release of transmitters, and the like—and longer grains for low-frequency synchronization, the induction of plastic changes, and so on. But again, while these faster and slower time scales are critical for understanding how the system works, only one time scale matters intrinsically—from the perspective of the conscious subject. Accordingly, IIT predicts that experience should only change if there is a change in the state of intrinsic units at their intrinsic update grain. Any other changes will affect the brain, but not experience. Even more stringently, the requirement that intrinsic units have binary macro states implies that any change in their micro state that does not translate into a switch of their macro state will not affect experience. For example, changes in the timing of neuronal firing, or in the rate of firing, may have clear-cut effects on the rest of the brain, but if they map onto the same intrinsic macro state, they will not have effects on the experience.

For IIT, physical existence is cause-effect power, with no need for primitive categorical properties. IIT’s analysis of cause-effect power starts from a causal model of a micro-physical substrate, which is defined by its transition probabilities, $\mathcal{T}_{U}$ Equation (1). The substrate TPM is taken to be a complete description of the substrate. The examples presented were analyzed based on the assumption that the substrate TPM was fully known and strictly stationary, with the goal of demonstrating the self-consistency of IIT’s approach and highlighting some of its consequences. In practice, the determination of complexes and their intrinsic units cannot be based on a full knowledge of the microphysical TPM. At most, the theoretical principles outlined here can serve as heuristic guidance for pointing to candidate complexes and intrinsic units, at the expense of many assumptions and approximations. Stationarity of the TPM over macro states is also merely a convenient assumption. In general, $\mathcal{T}_{U}$ is expected to evolve at every update, in accordance with IIT’s *principle of becoming* (“powers become what powers do,” to be considered in future work (Tononi forthcoming)).

## Supplementary Material

IntrinsicUnits_Supplement_niag013

## Data Availability

No new data were generated or analysed in support of this research. All code used to obtain figures and examples can be found at (Findlay and Marshall, 2025).
