# Supplementary material for: Intrinsic units: identifying a system’s causal grain
Source: Neurosci Conscious. 2026 Apr 15;2026(1):niag013. doi: 10.1093/nc/niag013 (PMC13082400; doi:10.1093/nc/niag013)
Supplement: IntrinsicUnits_Supplement_niag013 [file intrinsicunits_supplement_niag013.pdf]

Intrinsic Units: Identifying a system's causal grain — Supplementary material.

William Marshall[1*†], Graham Findlay[2,3†], Larissa Albantakis[2], Giulio Tononi[2*]

1 Department of Mathematics and Statistics, Brock University, St. Catharines, Ontario, Canada
2 Department of Psychiatry, University of Wisconsin, Madison, Wisconsin, United States of America
3 Neuroscience Training Program, University of Wisconsin, Madison, Wisconsin, United States of America

†These authors contributed equally to this work.
* Corresponding authors: wmarshall@brocku.ca, gtononi@wisc.edu

Supplement: Figures

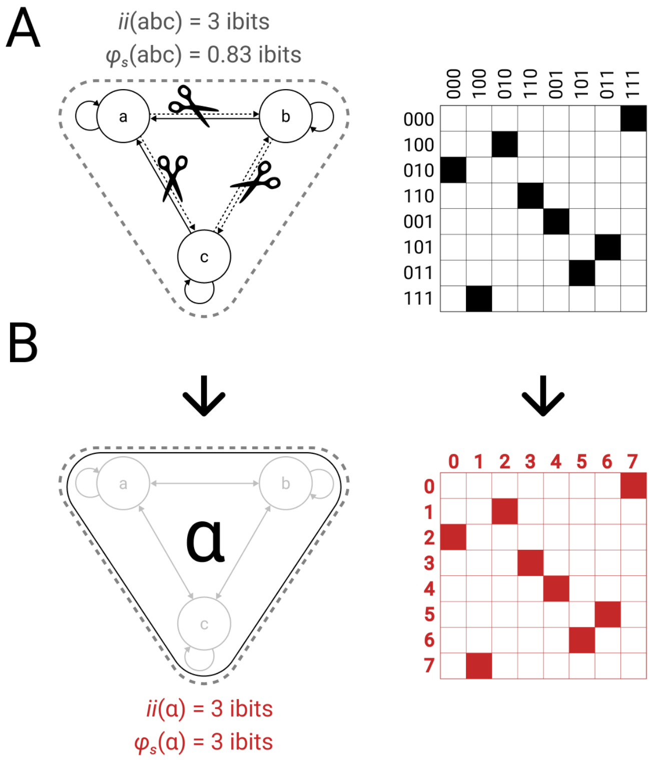

Figure 1: Binary units. (**A**) System $\{A, B, C\}$ is "causally perfect", meaning that every state has exactly one future state, and exactly one past state. It has the maximum possible intrinsic information (ii), 3 ibits, in every state. How much integrated information (φ_s) it has depends on its current state and how different cuts impact its transition probability matrix (right). In state $(0, 0, 0)$ the minimum information partition cuts the connections indicated by dotted black lines, and $\varphi_s(\text{abc}) = 0.83$ ibits. It has greater φ_s than any of its subsets (not shown), establishing $\{A, B, C\}$ in state $(0, 0, 0)$ as an admissible macro unit. (**B**) Let $\alpha = \{(A, B, C), (A, B, C), \tau_\alpha = 1, g_\alpha)\}$ be a candidate macro unit. If we allow arbitrary mappings from the state of $\{A, B, C\}$ to a non-binary state of α, then consider the mapping g_α where α has eight states, one for each microstate of $\{A, B, C\}$. The corresponding macro system $\{\alpha\}$ has 3 ibits of intrinsic information — no information was lost in the mapping from micro to macro. Moreover, since this system consists of a single unit it is irreducible (unit-ary) by definition, all its intrinsic information is integrated information, so $\varphi_s(\alpha) = 3$ ibits. Our putatively macro information and its structure is in fact all micro information and structure — none of it is intrinsic to the macro grain. It is a contradiction that all of the macro TPM's information and structure (the two are inseparable) is "hidden" from perturbation, observation, and partition at the macro grain, but there is a deeper issue too that this example makes manifest — that non-binary "units" at one grain imply the existence of internal mechanisms at another grain — which distinguish between and structure the macro states, and contribute to putatively macro cause-effect power, violating the strict separation of levels that is the premise of pitting the micro against the macro.

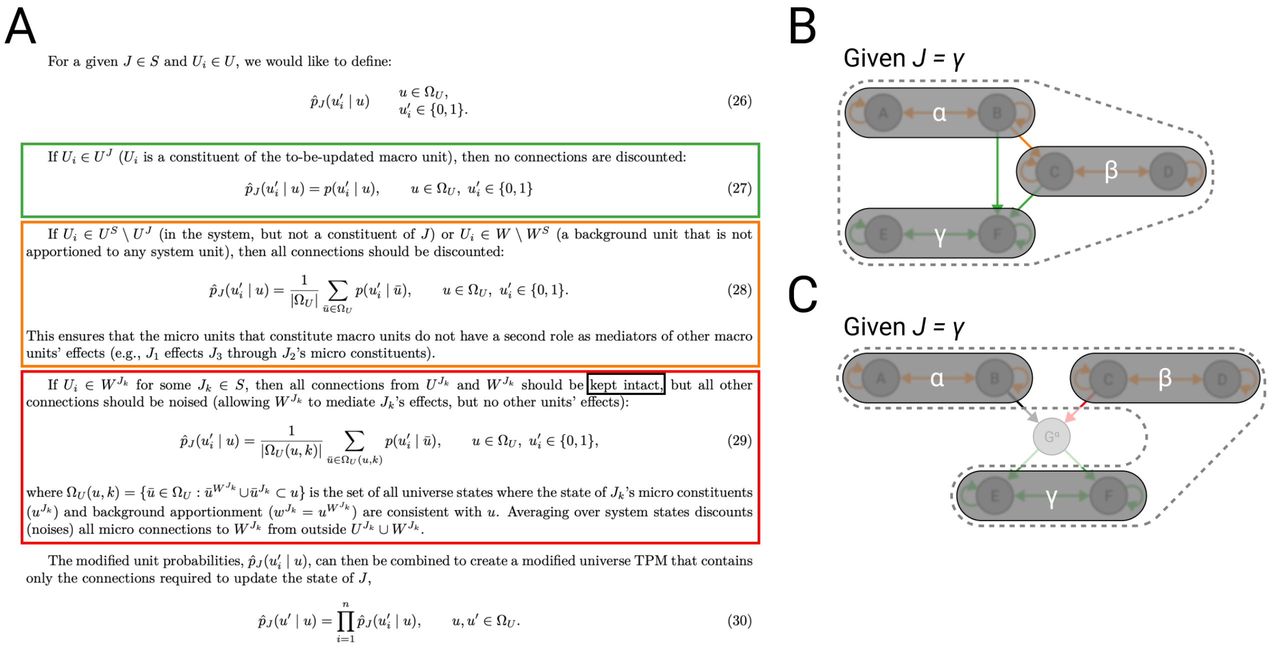
A

For a given $J \in S$ and $U_i \in U$, we would like to define:

$$\hat{p}_J(u_i' \mid u) \qquad \begin{array}{l} u \in \Omega_U, \\ u_i' \in \{0,1\}. \end{array} \tag{26}$$

If $U_i \in U^J$ (U_i is a constituent of the to-be-updated macro unit), then no connections are discounted:

$$\hat{p}_J(u_i' \mid u) = p(u_i' \mid u), \qquad u \in \Omega_U,\ u_i' \in \{0,1\} \tag{27}$$

If $U_i \in U^S \setminus U^J$ (in the system, but not a constituent of J) or $U_i \in W \setminus W^S$ (a background unit that is not apportioned to any system unit), then all connections should be discounted:

$$\hat{p}_J(u_i' \mid u) = \frac{1}{|\Omega_U|} \sum_{\bar{u} \in \Omega_U} p(u_i' \mid \bar{u}), \qquad u \in \Omega_U,\ u_i' \in \{0,1\}. \tag{28}$$

This ensures that the micro units that constitute macro units do not have a second role as mediators of other macro units' effects (e.g., J_1 effects J_3 through J_2's micro constituents).

If $U_i \in W^{J_k}$ for some $J_k \in S$, then all connections from U^{J_k} and W^{J_k} should be kept intact, but all other connections should be noised (allowing W^{J_k} to mediate J_k's effects, but no other units' effects):

$$\hat{p}_J(u_i' \mid u) = \frac{1}{|\Omega_U(u,k)|} \sum_{\bar{u} \in \Omega_U(u,k)} p(u_i' \mid \bar{u}), \qquad u \in \Omega_U,\ u_i' \in \{0,1\}, \tag{29}$$

where $\Omega_U(u,k) = \{\bar{u} \in \Omega_U : \bar{u}^{W^{J_k}} \cup \bar{u}^{J_k} \subset u\}$ is the set of all universe states where the state of J_k's micro constituents (u^{J_k}) and background apportionment ($w^{J_k} = u^{W^{J_k}}$) are consistent with u. Averaging over system states discounts (noises) all micro connections to W^{J_k} from outside $U^{J_k} \cup W^{J_k}$.

The modified unit probabilities, $\hat{p}_J(u_i' \mid u)$, can then be combined to create a modified universe TPM that contains only the connections required to update the state of J,

$$\hat{p}_J(u' \mid u) = \prod_{i=1}^{n} \hat{p}_J(u_i' \mid u), \qquad u, u' \in \Omega_U. \tag{30}$$

Figure 2: No double-counting of cause-effect power. (**A**) Eqs. 26–30 are reproduced from the main text. Colors indicates the implications of each Eq. for connections between micro units in U, when computing the modified transition probability matrix (TPM) for a given macro unit J. (**B**) System $S = \{\alpha, \beta, \gamma\}$ constituted of micro units $U = \{A, B, C, D, E, F\}$ has an update grain of $\tau = 2$. When computing the modified unit probabilities of γ, the connections shown in green are left intact (per Eq. 27), the connections shown in orange are noised (per Eq. 28), and Eq. 29 does not apply because there is no background apportionment. Why are these procedures important? When computing the modified unit probabilities of γ, we must not double-count the causal power of C: in β's direct effect on γ, and indirectly mediating α's effect on γ. When considering the update TPM for γ, this is accomplished by noising the micro connection from B to C (per Eq. 28). (**C**) System $S = \{\alpha, \beta, \gamma\}$ constituted of micro units $U = \{A, B, C, D, E, F, G^\alpha\}$ has an update grain of $\tau = 2$. The superscript on G^α indicates that G is part of α's apportionment. When computing the modified unit probabilities of γ, the connections shown in green and black are left intact (per Eqs. 27 and 29, respectively), and the connections shown in orange and red are noised (per Eqs. 28 and 29, respectively). Why are these procedures important? When computing the modified unit probabilities of γ, we must not double-count the causal power of G mediating both α and β's effects on γ. This is accomplished by noising the micro connection from C to G (per Eq. 29).

Supplement: Comparison with frameworks for quantifying emergence

Emergence has been variously defined, including as the production of complex dynamics from simple interactions, and as the production of a whole that is "more than the sum" of its parts. Often a distinction is made between nominal, weak, and strong emergence: Nominal emergence refers to macroscale properties, patterns, or entities that add only descriptive convenience (e.g., a line emerges from the points that constitute it); weak emergence refers to macro-properties that are surprising or computationally irreducible, yet fully determined by microscale dynamics (e.g., in Conway's Game of Life, gliders and oscillators are surprising and unpredictable except by simulation, but fully determined by the game's simple rules); strong emergence refers to macroscale entities or patterns with genuinely novel properties that are not even in-principle reducible to properties of the microscale (Bedau 1997). It has been suggested that consciousness is one example of strong emergence (Chalmers 1996). Even if that notion is rejected, both consciousness and neural dynamics might be construed as weakly emergent (Seth 2010), and so the science of consciousness and efforts to formalize emergence have a shared history.

A distinction can also be drawn between ontological emergence, in which new things come into being, and epistemic or explanatory emergence, in which macroscales simply offer gains in description, prediction, or explanation over microscales. Often, ontological emergence is equated with strong emergence, and epistemic or explanatory emergence with weak emergence (Bedau 2002; O'Connor 2021) (but see (Carroll and Parola 2024)). Because description, prediction, and explanation are obviously useful, most frameworks for quantifying emergence (McKenzie 2025) are concerned only with the latter (Shalizi and Moore 2003; Gershenson and Fernández 2012; Polani 2006; Seth 2010; Pfante et al. 2014; Wolpert et al. 2014; Marchese et al. 2022).

Causal emergence is a technical term referring to the specific phenomenon in which a macro system has greater causal power than the micro system on which it supervenes (Hoel et al. 2013; Yuan et al. 2024), where A supervenes on B if any change in A necessarily entails a change in B (Stalnaker 1996). Both weak and strong emergence have been defined in terms of the causal relationship between macro and micro (Bedau 2002; O'Connor 2021): What makes a macro-level entity genuinely emergent or existent is not just that it offers a convenient description, but that it makes a causal difference, and one that can exclude (or at least not be excluded by) micro-causes (List et al. 2009). Thus, there is a strong argument that any measure of emergence should be causal in nature (Seth 2010; Hoel et al. 2013; Yuan et al. 2024). Causation, in turn, is closely related to information: the former's core primitives (necessity and sufficiency) are almost automatically formulated in term of the latter's (determinism and degeneracy) (Comolatti et al. 2025), and causal systems can be viewed "as passing information from the past to the future over the channel of the present" (Varley and Hoel 2022). For this reason, nearly all frameworks for causal emergence share the language of information theory (Yuan et al. 2024).

Given the close association of both emergence and existence with cause-effect power, it should come as no surprise that IIT's framework for identifying intrinsic existence at macro grains resembles a framework for causal emergence. Indeed, the initial development of a rigorous, quantitative theory of causal emergence (Hoel et al. 2013) was motivated in part by the need to identify intrinsic existence at macro grains (Hoel et al. 2016; Marshall et al. 2018). However, although IIT's framework involves measuring causal power at several scales ("grains"), and assumes that causal power can be higher at macro scales than at micro ones, IIT is not an emergentist theory of consciousness in anything but a technical sense. Instead, the relationship of parts to wholes in IIT (e.g., of micro units to macro units, or units to complexes) is more akin to philosophical positions in which the traditional primacy of parts (in the sense of being more "fundamental" than wholes) is flipped on its head: Parts do not exist except as constituents of wholes, and are not, ontologically speaking, precursors to anything (Chis-Ciure et al. 2024).

Nevertheless, because of the deep connection between IIT and causal emergence in the technical sense, and because it has inspired causal emergence frameworks (Hoel et al. 2013; Mediano et al. 2019; Rosas et al. 2020; Grasso et al. 2021; Sampson 2024), a comparison with alternative frameworks for quantifying emergence is useful. It is important to keep in mind that differences between frameworks follow from differences in purpose and in underlying model assumptions. Whereas IIT is primarily concerned with consciousness (intrinsic existence) and the identification of intrinsic, irreducible cause-effect power as dictated by the postulates, other frameworks will have other objectives. For example, their interests may be in identifying macroscale descriptions of systems that improve prediction (Crutchfield 1994; Shalizi and Moore 2003; Rosas et al. 2020) or explanation (Marrow et al. 2020; Klein et al. 2021; Zhang and Liu 2022), achieving dimensionality reduction or descriptive compression while preserving underlying microscale dynamics (Klein and Hoel 2020; Rosas et al. 2024; Zhang et al. 2025), or finding closed levels of description (Chang et al. 2020; Rosas et al. 2024).

Our framework is similar in spirit to those that identify macro grains with maxima of self-determination (Bertschinger et al. 2008; Seth 2010; Barnett and Seth 2023; Rosas et al. 2024; Milinkovic et al. 2025), if self-determination is understood as intrinsic existence (Albantakis et al. 2019). Such self-determination has also been related to concepts like "autonomy" or "closedness" (Varela 1979). Our framework is also similar in spirit to those that assert the plausibility of a compositional, observer-independent information structure within a system, and consider that structure to be an important determinant in whether and how a macroscale emerges (Polani 2006).

Frameworks differ widely in their notions of scale and grain. In some frameworks, a macroscale is defined by a grain size, which is assumed or required to be homogeneous (Hoel et al. 2013). If the underlying model is not a graph or network, the requirement for homogeneity at a given scale can take different forms depending on the framework. For example, one could require that all macrostates or variables (e.g., renormalization groups) have the same number of microstates or variables (Bar-Yam 2004; Blundell 2019), or that constituents of a macro variable are predictively equivalent (Shalizi and Crutchfield 2001). On the other end of the spectrum is a framework that allows for heterogeneous grains within the same macro system, considers superimposed macro systems at every "scale", and apportions out some degree of causation to each, without any single scale excluding the others (Hoel 2025). Although they must contend with philosophical issues of overdetermination, causal exclusion, and inter-scale causation (List et al. 2009), fully multiscale frameworks may be especially well-suited for describing biological systems, where it has been suggested that no preferred scale exists (a phenomenon termed "a-mergence") (Noble 2012; Noble and Noble 2019).

Our framework shares much in common with the multi-scale approach, both in its allowance for heterogeneous unit grains within a complex, and in its hierarchical construction of macro units from meso and micro units. However, a key difference between the extreme multiscale approach and IIT stems from the exclusion postulate, which implies that two substrates of consciousness cannot overlap, nor can two macro units overlap within the same substrate. In IIT, whether a macro system is built up in levels, which precise macro and meso units it is built from, and which mappings define those units' state, are all ultimately determined by which units satisfy the postulates while maximizing φ at the top level of the hierarchy (i.e., of the complex). Thus, there is always a reason why a complex and its intrinsic units are precisely what they are: the principle of maximal existence.

Frameworks also differ in whether or not they provide a means of identifying one or more scales of interest. Some frameworks, like ours, perform an exhaustive search across scales, and pick the one that maximizes some criterion (Hoel et al. 2013), while others estimate the result of a search across scales (Griebenow et al. 2019; Zhang and Liu 2022; Zhang et al. 2025). Other frameworks may be more interested with describing macroscale systems at some user-selected scale (Mediano et al. 2019), or simply defer the question.

In some frameworks, particularly those influenced by statistical physics, it is expected that macro variables can be obtained through coarse-graining (Saunders and Voth 2013; Wolpert et al. 2014). It is less common to find a framework like ours, that can also accomodate macro variables obtained through black-boxing. As discussed in section 3.3, black boxes correspond to the typical notion of macro units in the special sciences, because they are constituted of heterogeneous micro units that are often compartmentalized and have highly specific functions, which would be muddled by averaging (Marshall et al. 2018).

In our framework, the underlying model is a discrete-valued stochastic model for a physical universe of interacting units with binary states, assumed to be a complete causal network without instantaneous causation. [1] In IIT, all measures of cause-effect power applied to this model are state-dependent. That is, any time the microstate of the physical universe changes, IIT's identified complexes and their intrinsic units are subject to change. To our knowledge, all other emergence frameworks are process-level: they would identify and quantify emergence based on the system's TPM alone (without considering the system's current state), or its representation as a Markov chain, stationary distribution, etc. IIT's unique state-dependency is because it is exclusively concerned with consciousness, which only ever exists here and now, even if its contents include, for example, the feeling of temporal flow (Comolatti and Hoel 2025).

Process-level frameworks are also, unlike IIT, concerned with the dynamical consistency of macro and micro

[1] Since, with the preceding state u held fixed, nothing instantaneous at the current state u' can jointly inform u'_i, there is no mutual information—and partial information decompositions will find zero "synergistic" information (Williams and Beer 2010)—between units within a single state of U, once the past is controlled for. That is, if $Y = u'_i$ and $X \subseteq u' \setminus u'_i$, then $I(Y; X \mid u) = 0$ and $\text{Synergy}(Y; X \mid u) = 0$. On the other hand, there is still room for synergy between units from one state to the next, realized by higher-order mechanisms (Barbosa et al. 2021) already encoded in $\mathcal{T}_U$: no extra-TPM causal power is posited in such cases.

scales. The reasoning behind consistency requirements is essentially that (i) a macroscale should be a (good) model of the microscale, and that (ii) the model is no good if its temporal evolution would diverge from the microscale's. In some cases, it is even required that the micro-to-macro operation (e.g., coarse-graining) and state transitions should be fully commutative (Pfante et al. 2014). Markovian frameworks (e.g., (Rosas et al. 2024)) may enforce consistency by requiring that macrostates satisfy lumpability—a partition of the state space is lumpable if, for any two microstates i, j in the same macrostate, the total transition probability from i to any other macrostate M is the same as the total transition probability from j into M—thereby ensuring that the macro system is itself Markovian (Kemeny and Snell 1983). This is a fairly strong requirement, and other criteria for evaluating consistency exist (Rubenstein et al. 2017; Klein and Hoel 2020). Although high consistency between micro and macro scales generally implies good predictive power of the macro model over extended periods, it may come at the cost of model simplicity and achievable compression. In principle this tradeoff could be set by a tunable parameter, though more often some objective function that combines both is optimized (e.g., (Wolpert et al. 2014)).

Because our framework is not process-oriented, and is concerned with the characterization of immediate, intrinsic, cause-effect power rather than dynamics, we eschew a requirement for dynamical consistency in favor of a requirement for consistency with the postulates. Our macro units, derived via causal marginalization, will often not satisfy traditional Markov properties under coarse-graining, but instead must have cause-effect power that is intrinsic, specific, irreducible, definite, and structured. In Figure 2A, for example, the candidate macro units shown satisfy lumpability, but we do not consider them a good "model" of what actually exists, because they hide a total lack of integrated cause-effect power.

Finally, while many frameworks are formulated for discrete-valued variables, a number of recent approaches have been developed to address continuous systems (Mediano et al. 2019; Rosas et al. 2020; Zhang and Liu 2022; Rupe and Crutchfield 2023; Sampson 2024). These methods vary in scope: some are formulated in terms of mutual information (which has limitations when applied to matters of causal emergence (Hoel 2025)), place less emphasis on strictly causal emergence, require pre-defined macro variables, or focus on sufficient (rather than necessary) conditions for emergence (Yuan et al. 2024). At the same time, these design choices make them well suited for direct application to neural time series data (Mediano et al. 2022). Our framework, by contrast, assumes a complete causal model with binary micro units and discrete state transitions, so applying it to continuous data would require discretization. Although simple binarization of continuous variables has been explored (Haun et al. 2017; Afrasiabi et al. 2021), more principled discretization methods (e.g., (Varley 2020)) that yield accurate estimates of integrated information are an important future direction for research. See (Barrett and Seth 2011; Oizumi et al. 2016; Gomez et al. 2021) for related efforts to extend integrated information measures from IIT 2.0 and 3.0 to non-binary systems, and (Sampson 2024) for a general approach to deriving causal models—macro from micro, and discrete from continuous.

References

Afrasiabi, M. et al. (Apr. 2021). "Consciousness Depends on Integration between Parietal Cortex, Striatum, and Thalamus". In: *Cell Systems* 12.4, 363–373.e11. ISSN: 24054712. DOI: 10.1016/j.cels.2021.02.003. URL: https://linkinghub.elsevier.com/retrieve/pii/S2405471221000776 (visited on 08/21/2025).

Albantakis, L. et al. (May 2019). "What Caused What? A Quantitative Account of Actual Causation Using Dynamical Causal Networks". In: *Entropy* 21.5 (5), p. 459. ISSN: 1099-4300. DOI: 10.3390/e21050459. URL: https://www.mdpi.com/1099-4300/21/5/459 (visited on 07/28/2024).

Bar-Yam, Y. (July 2004). "A Mathematical Theory of Strong Emergence Using Multiscale Variety". In: *Complexity* 9.6, pp. 15–24. ISSN: 1076-2787, 1099-0526. DOI: 10.1002/cplx.20029. URL: https://onlinelibrary.wiley.com/doi/10.1002/cplx.20029 (visited on 08/19/2025).

Barbosa, L. S. et al. (Mar. 2021). "Mechanism Integrated Information". In: *Entropy* 23.3 (3), p. 362. ISSN: 1099-4300. DOI: 10.3390/e23030362. URL: https://www.mdpi.com/1099-4300/23/3/362 (visited on 07/31/2024).

Barnett, L. and A. K. Seth (July 17, 2023). "Dynamical Independence: Discovering Emergent Macroscopic Processes in Complex Dynamical Systems". In: *Physical Review E* 108.1, p. 014304. ISSN: 2470-0045, 2470-0053. DOI: 10.1103/PhysRevE.108.014304. URL: https://link.aps.org/doi/10.1103/PhysRevE.108.014304 (visited on 08/20/2025).

Barrett, A. B. and A. K. Seth (Jan. 2011). "Practical Measures of Integrated Information for Time-Series Data." In: *PLoS computational biology* 7.1, e1001052. ISSN: 1553-7358. DOI: 10.1371/journal.pcbi.1001052. PMID: 21283779. URL: http://www.pubmedcentral.nih.gov/articlerender.fcgi?artid=3024259%7B&%7Dtool=pmcentrez%7B&%7Drendertype=abstract.

Bedau, M. A. (1997). "Weak Emergence". In: *Philosophical Perspectives* 11, pp. 375–399. DOI: 10.1111/0029-4624.31.s11.17.

— (2002). "Downward Causation and the Autonomy of Weak Emergence". In: *Principia* 6.1, pp. 5–50.

Bertschinger, N. et al. (Feb. 2008). "Autonomy: An Information Theoretic Perspective". In: *Biosystems* 91.2, pp. 331–345. ISSN: 03032647. DOI: 10.1016/j.biosystems.2007.05.018. URL: https://linkinghub.elsevier.com/retrieve/pii/S0303264707001037 (visited on 08/19/2025).

Blundell, S. J. (2019). "Phase Transitions, Broken Symmetry and the Renormalization Group". In: *The Routledge Handbook of Emergence*. Ed. by S. C. Gibb, R. F. Hendry, and T. Lancaster. London New York: Routledge. ISBN: 978-1-315-67521-3.

Carroll, S. M. and A. Parola (Oct. 20, 2024). *What Emergence Can Possibly Mean*. DOI: 10.48550/arXiv.2410.15468. arXiv: 2410.15468 [physics]. URL: http://arxiv.org/abs/2410.15468 (visited on 09/10/2025). Pre-published.

Chalmers, D. J. (1996). *The Conscious Mind. In Search of a Fundamental Theory*. Oxford University Press.

Chang, A. Y. C. et al. (July 15, 2020). "Information Closure Theory of Consciousness". In: *Frontiers in Psychology* 11, p. 1504. ISSN: 1664-1078. DOI: 10.3389/fpsyg.2020.01504. URL: https://www.frontiersin.org/article/10.3389/fpsyg.2020.01504/full (visited on 08/19/2025).

Chis-Ciure, R. et al. (June 2024). *FAQ: Is IIT an Emergentist Theory of Consciousness?* IIT Wiki. URL: https://www.iit.wiki/faqs/philosophy,%20doi:10.5281/zenodo.14160283.

Comolatti, R., M. Grasso, and G. Tononi (Oct. 17, 2025). "Why Does Time Feel the Way It Does? Toward a Principled Account of Temporal Experience". In: *iScience* 28.10, p. 113434. ISSN: 2589-0042. DOI: 10.1016/j.isci.2025.113434. URL: https://www.sciencedirect.com/science/article/pii/S2589004225016955 (visited on 09/22/2025).

Comolatti, R. and E. Hoel (Aug. 2025). "Consilience in Causation: Causal Emergence Is Found Across Measures of Causation". In: *Entropy* 27.8, p. 825. ISSN: 1099-4300. DOI: 10.3390/e27080825. URL: https://www.mdpi.com/1099-4300/27/8/825 (visited on 08/08/2025).

Crutchfield, J. P. (Aug. 1994). "The Calculi of Emergence: Computation, Dynamics and Induction". In: *Physica D: Nonlinear Phenomena* 75.1–3, pp. 11–54. ISSN: 01672789. DOI: 10.1016/0167-2789(94)90273-9. URL: https://linkinghub.elsevier.com/retrieve/pii/0167278994902739 (visited on 08/19/2025).

Gershenson, C. and N. Fernández (Nov. 2012). "Complexity and Information: Measuring Emergence, Self-organization, and Homeostasis at Multiple Scales". In: *Complexity* 18.2, pp. 29–44. ISSN: 1076-2787, 1099-0526. DOI: 10.1002/cplx.21424. URL: https://onlinelibrary.wiley.com/doi/10.1002/cplx.21424 (visited on 08/19/2025).

Gomez, J. D. et al. (Dec. 2021). "Computing Integrated Information (Φ) in Discrete Dynamical Systems with Multi-Valued Elements". In: *Entropy 2021, Vol. 23, Page 6* 23.1, p. 6. DOI: 10.3390/e23010006. URL: https://dx.doi.org/10.3390/e23010006.

Grasso, M. et al. (Oct. 2021). "Causal Reductionism and Causal Structures". In: *Nature Neuroscience* 24.10, pp. 1348–1355. ISSN: 15461726. DOI: 10.1038/s41593-021-00911-8. URL: https://www.nature.com/articles/s41593-021-00911-8.

Griebenow, R., B. Klein, and E. Hoel (2019). *Finding the Right Scale of a Network: Efficient Identification of Causal Emergence through Spectral Clustering*. Version 2. DOI: 10.48550/ARXIV.1908.07565. URL: https://arxiv.org/abs/1908.07565 (visited on 08/20/2025). Pre-published.

Haun, A. M. et al. (Sept. 2017). "Conscious Perception as Integrated Information Patterns in Human Electrocorticography". In: *eneuro* 4.5, ENEURO.0085–17.2017. ISSN: 2373-2822. DOI: 10.1523/ENEURO.0085-17.2017. URL: https://www.eneuro.org/lookup/doi/10.1523/ENEURO.0085-17.2017 (visited on 08/21/2025).

Hoel, E. (Apr. 21, 2025). *Causal Emergence 2.0: Quantifying Emergent Complexity*. DOI: 10.48550/arXiv.2503.13395. arXiv: 2503.13395 [cs]. URL: http://arxiv.org/abs/2503.13395 (visited on 08/06/2025). Pre-published.

Hoel, E. P., L. Albantakis, and G. Tononi (Dec. 3, 2013). "Quantifying Causal Emergence Shows That Macro Can Beat Micro". In: *Proceedings of the National Academy of Sciences* 110.49, pp. 19790–19795. ISSN: 0027-8424, 1091-6490. DOI: 10.1073/pnas.1314922110. URL: https://pnas.org/doi/full/10.1073/pnas.1314922110 (visited on 07/30/2024).

Hoel, E. P. et al. (2016). "Can the Macro Beat the Micro? Integrated Information across Spatiotemporal Scales". In: *Neuroscience of Consciousness* 2016.1, niw012. ISSN: 2057-2107. DOI: 10.1093/nc/niw012. URL: https://academic.oup.com/nc/article-lookup/doi/10.1093/nc/niw012 (visited on 08/19/2025).

Kemeny, J. G. and J. L. Snell (1983). *Finite Markov Chains*. Undergraduate Texts in Mathematics. New York: Springer-Verlag. 224 pp. ISBN: 978-0-387-90192-3.

Klein, B. and E. Hoel (Apr. 4, 2020). “The Emergence of Informative Higher Scales in Complex Networks”. In: *Complexity* 2020, pp. 1–12. ISSN: 1076-2787, 1099-0526. DOI: 10.1155/2020/8932526. URL: https://www.hindawi.com/journals/complexity/2020/8932526/ (visited on 08/19/2025).

Klein, B. et al. (Dec. 31, 2021). “Evolution and Emergence: Higher Order Information Structure in Protein Interactomes across the Tree of Life”. In: *Integrative Biology* 13.12, pp. 283–294. ISSN: 1757-9708. DOI: 10.1093/intbio/zyab020. URL: https://academic.oup.com/ib/article/13/12/283/6474503 (visited on 08/20/2025).

List, C., P. Menzies, and Journal of Philosophy, Inc. (2009). “Nonreductive Physicalism and the Limits of the Exclusion Principle”. In: *Journal of Philosophy* 106.9, pp. 475–502. ISSN: 0022-362X. DOI: 10.5840/jphil2009106936. URL: http://www.pdcnet.org/oom/service?url_ver=Z39.88-2004&rft_val_fmt=&rft.imuse_id=jphil_2009_0106_0009_0475_0502&svc_id=info:www.pdcnet.org/collection (visited on 08/19/2025).

Marchese, E., G. Caldarelli, and T. Squartini (May 30, 2022). “Detecting Mesoscale Structures by Surprise”. In: *Communications Physics* 5.1, p. 132. ISSN: 2399-3650. DOI: 10.1038/s42005-022-00890-7. URL: https://www.nature.com/articles/s42005-022-00890-7 (visited on 08/19/2025).

Marrow, S., E. J. Michaud, and E. Hoel (Dec. 18, 2020). “Examining the Causal Structures of Deep Neural Networks Using Information Theory”. In: *Entropy* 22.12, p. 1429. ISSN: 1099-4300. DOI: 10.3390/e22121429. URL: https://www.mdpi.com/1099-4300/22/12/1429 (visited on 08/20/2025).

Marshall, W., L. Albantakis, and G. Tononi (Apr. 23, 2018). “Black-Boxing and Cause-Effect Power”. In: *PLOS Computational Biology* 14.4, e1006114. ISSN: 1553-7358. DOI: 10.1371/journal.pcbi.1006114. URL: https://journals.plos.org/ploscompbiol/article?id=10.1371/journal.pcbi.1006114 (visited on 09/12/2018).

McKenzie, R. H. (2025). *Emergence: From Physics to Biology, Sociology, and Computer Science*. Version 1. DOI: 10.48550/ARXIV.2508.08548. URL: https://arxiv.org/abs/2508.08548 (visited on 08/19/2025). Pre-published.

Mediano, P. A. M. et al. (Sept. 2019). “Beyond Integrated Information: A Taxonomy of Information Dynamics Phenomena”. In: *arXiv* 1909.02297. URL: http://arxiv.org/abs/1909.02297.

Mediano, P. A. M. et al. (May 23, 2022). “Greater than the Parts: A Review of the Information Decomposition Approach to Causal Emergence”. In: *Philosophical Transactions of the Royal Society A: Mathematical, Physical and Engineering Sciences* 380.2227, p. 20210246. DOI: 10.1098/rsta.2021.0246. URL: https://royalsocietypublishing.org/doi/10.1098/rsta.2021.0246 (visited on 07/31/2024).

Milinkovic, B. et al. (May 12, 2025). “Capturing the Emergent Dynamical Structure in Biophysical Neural Models”. In: *PLOS Computational Biology* 21.5, e1012572. ISSN: 1553-7358. DOI: 10.1371/journal.pcbi.1012572. URL: https://journals.plos.org/ploscompbiol/article?id=10.1371/journal.pcbi.1012572 (visited on 03/01/2026).

Noble, D. (Feb. 6, 2012). “A Theory of Biological Relativity: No Privileged Level of Causation”. In: *Interface Focus* 2.1, pp. 55–64. ISSN: 2042-8898, 2042-8901. DOI: 10.1098/rsfs.2011.0067. URL: https://royalsocietypublishing.org/doi/10.1098/rsfs.2011.0067 (visited on 08/19/2025).

Noble, R. and D. Noble (2019). “A-Mergence of Biological Systems”. In: *The Routledge Handbook of Emergence*. Ed. by S. C. Gibb, R. F. Hendry, and T. Lancaster. London New York: Routledge. ISBN: 978-1-315-67521-3.

O’Connor, T. (2021). “Emergent Properties”. In: *The Stanford Encyclopedia of Philosophy*. Ed. by E. N. Zalta. Winter 2021. Metaphysics Research Lab, Stanford University. URL: https://plato.stanford.edu/archives/win2021/entries/properties-emergent/ (visited on 08/19/2025).

Oizumi, M. et al. (Jan. 2016). “Measuring Integrated Information from the Decoding Perspective”. In: *PLOS Computational Biology* 12.1, e1004654. ISSN: 1553-7358. DOI: 10.1371/journal.pcbi.1004654. URL: http://journals.plos.org/ploscompbiol/article?id=10.1371/journal.pcbi.1004654.

Pfante, O. et al. (Mar. 2014). “Comparison Between Different Methods of Level Identification”. In: *Advances in Complex Systems* 17.02, p. 1450007. ISSN: 0219-5259, 1793-6802. DOI: 10.1142/S0219525914500076. URL: https://www.worldscientific.com/doi/abs/10.1142/S0219525914500076 (visited on 08/19/2025).

Polani, D. (2006). “Emergence, Intrinsic Structure of Information, and Agenthood”. In: *Complex Systems* 1937. ISSN: 1081-0625.

Rosas, F. E. et al. (Dec. 21, 2020). “Reconciling Emergences: An Information-Theoretic Approach to Identify Causal Emergence in Multivariate Data”. In: *PLOS Computational Biology* 16.12. Ed. by D. Marinazzo, e1008289. ISSN: 1553-7358. DOI: 10.1371/journal.pcbi.1008289. URL: https://dx.plos.org/10.1371/journal.pcbi.1008289 (visited on 08/19/2025).

Rosas, F. E. et al. (2024). *Software in the Natural World: A Computational Approach to Hierarchical Emergence*. Version 2. DOI: 10.48550/ARXIV.2402.09090. URL: https://arxiv.org/abs/2402.09090 (visited on 08/19/2025). Pre-published.

Rubenstein, P. K. et al. (July 2017). “Causal Consistency of Structural Equation Models”. arXiv: 1707.00819. URL: http://arxiv.org/abs/1707.00819.

Rupe, A. T. and J. P. Crutchfield (2023). *On Principles of Emergent Organization*. Version 1. DOI: 10.48550/ARXIV.2311.13749. URL: https://arxiv.org/abs/2311.13749 (visited on 08/21/2025). Pre-published.

Sampson, J. T. (2024). "Integrated Information Theory of Consciousness in Conventional Computing". San Francisco State University. DOI: 10.46569/hq37vw99k. URL: https://doi.org/10.46569/hq37vw99k.

Saunders, M. G. and G. A. Voth (May 6, 2013). "Coarse-Graining Methods for Computational Biology". In: *Annual Review of Biophysics* 42.1, pp. 73–93. ISSN: 1936-122X, 1936-1238. DOI: 10.1146/annurev-biophys-083012-130348. URL: http://www.annualreviews.org/doi/10.1146/annurev-biophys-083012-130348 (visited on 08/20/2025).

Seth, A. K. (Apr. 1, 2010). "Measuring Autonomy and Emergence via Granger Causality". In: *Artificial Life* 16.2, pp. 179–196. ISSN: 1064-5462. DOI: 10.1162/artl.2010.16.2.16204. URL: https://doi.org/10.1162/artl.2010.16.2.16204 (visited on 08/06/2025).

Shalizi, C. R. and C. Moore (2003). "What Is a Macrostate? Subjective Observations and Objective Dynamics". Version 1. In: DOI: 10.48550/ARXIV.COND-MAT/0303625. URL: https://arxiv.org/abs/cond-mat/0303625 (visited on 08/19/2025).

Shalizi, C. R. and J. P. Crutchfield (2001). "Computational Mechanics: Pattern and Prediction, Structure and Simplicity". In: *Journal of statistical physics* 104.3–4, pp. 817–879. URL: http://www.springerlink.com/index/W21421233N715040.pdf.

Stalnaker, R. (1996). "Varieties of Supervenience". In: *Noûs* 30, p. 221. ISSN: 00294624. DOI: 10.2307/2216245. JSTOR: 2216245. URL: https://www.jstor.org/stable/2216245?origin=crossref (visited on 08/19/2025).

Varela, F. J. (1979). *Principles of Biological Autonomy*. North Holland. ISBN: 0-444-00321-5.

Varley, T. F. (2020). *Causal Emergence in Discrete and Continuous Dynamical Systems*. Version 1. DOI: 10.48550/ARXIV.2003.13075. URL: https://arxiv.org/abs/2003.13075 (visited on 08/21/2025). Pre-published.

Varley, T. F. and E. Hoel (May 23, 2022). "Emergence as the Conversion of Information: A Unifying Theory". In: *Philosophical Transactions of the Royal Society A: Mathematical, Physical and Engineering Sciences* 380.2227, p. 20210150. DOI: 10.1098/rsta.2021.0150. URL: https://royalsocietypublishing.org/doi/full/10.1098/rsta.2021.0150 (visited on 08/06/2025).

Williams, P. L. and R. D. Beer (Apr. 2010). "Nonnegative Decomposition of Multivariate Information". arXiv: 1004.2515. URL: http://arxiv.org/abs/1004.2515.

Wolpert, D. H. et al. (2014). *Optimal High-Level Descriptions of Dynamical Systems*. Version 2. DOI: 10.48550/ARXIV.1409.7403. URL: https://arxiv.org/abs/1409.7403 (visited on 08/19/2025). Pre-published.

Yuan, B. et al. (Feb. 2024). "Emergence and Causality in Complex Systems: A Survey of Causal Emergence and Related Quantitative Studies". In: *Entropy* 26.2 (2), p. 108. ISSN: 1099-4300. DOI: 10.3390/e26020108. URL: https://www.mdpi.com/1099-4300/26/2/108 (visited on 08/06/2025).

Zhang, J. and K. Liu (Dec. 23, 2022). "Neural Information Squeezer for Causal Emergence". In: *Entropy* 25.1, p. 26. ISSN: 1099-4300. DOI: 10.3390/e25010026. URL: https://www.mdpi.com/1099-4300/25/1/26 (visited on 08/20/2025).

Zhang, J. et al. (Jan. 25, 2025). "Dynamical Reversibility and a New Theory of Causal Emergence Based on SVD". In: *npj Complexity* 2.1, p. 3. ISSN: 2731-8753. DOI: 10.1038/s44260-025-00028-0. URL: https://www.nature.com/articles/s44260-025-00028-0 (visited on 08/20/2025).
